# Light-controlled genome editing by activation of Cas9-mRNA translation†

**DOI:** 10.1039/d5sc01999k

**Published:** 2025-06-30

**Authors:** Helena Schepers, Greta Charlotte Dahm, Martin Sumser, Sabine Hüwel, Andrea Rentmeister

**Affiliations:** a Institute of Biochemistry, University of Münster Corrensstr. 36 48149 Münster Germany; b Department of Chemistry, Ludwig-Maximilians-Universität München Butenandstr. 5–13 81377 Munich Germany a.rentmeister@lmu.de

## Abstract

Genome editing by the nuclease Cas9 and guide RNAs enables precise inactivation of genes and presents the basis for numerous research tools and emerging therapies. A critical aspect is the nuclease activity causing off-target effects. Approaches to control where and when active Cas9 is present are therefore desirable. Using Cas9-mRNA already presents a viable way to limit nuclease activity temporally but does not permit controlled induction. Here, we show that Cas9 activity is readily obtained by irradiation of cells transfected with a translationally muted Cas9-mRNA. Using a dual reporter system, we confirm light-mediated knockout of the eGFP-gene by flow cytometry, fluorescence microscopy, Western blotting and sequencing. This system does not involve photocaged proteins nor photocaged guide RNAs but relies on mRNA with a single photocleavable protecting group at the 5′ cap produced by *in vitro* transcription. This is the first demonstration of using light to activate muted Cas9-mRNA, leading to permanent alterations on the DNA level, despite the messenger itself being transient in nature.

## Abstract 2

Spatiotemporal control of Cas9-mediated genome editing by light using a cap-photocaged Cas9 mRNA.

## Graphical abstract

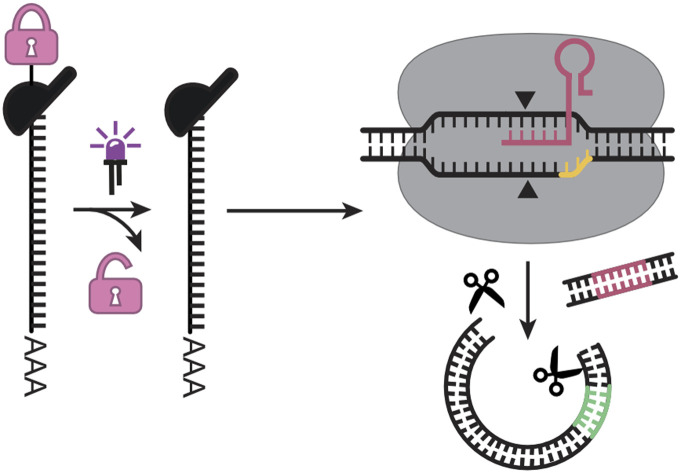

## Introduction

Genome editing by Cas9 or other nucleases has become a state-of-the-art tool to make knockout cell-lines and organisms. It bears potential for therapeutic applications and several clinical studies are underway. A key aspect in using nucleases is to control their action with high precision. This relates to both activating and deactivating the nuclease activity. Selective activation is important for synchronizing onset and targeting a subset of cells, while deactivation is important to avoid off-target effects.

To control the activity of genome editing, various approaches have been taken. For cell culture and model organisms, light is an excellent trigger as it can be controlled with high spatio-temporal precision.^1,2^ With respect to genome editing, which requires Cas9 protein and single guide RNAs (sgRNAs), light-control has been achieved by installing photocleavable protecting groups (PPG) at either one of these components. Genetic code expansion was used to control the activity of the Cas9 protein by light *via* a photocaged lysine important for the interaction with the sgRNA.^3^ The application in cells requires genetic introduction of the tRNA/tRNA synthetase pair. As an alternative approach, in many reports, sgRNAs were synthesized with multiple PPGs at the nucleobase or the sugar moiety.^3–6^ The positions of the PPGs along the sgRNA can be chosen to block hybridization to the target RNA. The placement of the PPGs next to the seed region still allowed Cas9 binding but prevented its activity until irradiation by light.^7^ In addition, alternative approaches involving PPGs on sgRNA (*e.g. via* photocleavable linkers) have been developed.^8–11^

To deactivate Cas9 function upon irradiation, sgRNAs with photocleavable linkers proved successful. These modified guide RNAs enhanced specificity of genome editing and allowed to synchronize DNA damage.^10^ A similar approach using the mRNA precursor of the nuclease has proven to be a viable option.^12^ Compared to DNA, mRNA does not need to enter the nucleus but is readily translated to the respective protein in the cytoplasm. Since the half-life of mRNA is typically in the range of hours, the nuclease is only produced for a limited time. However, regular Cas9-mRNA added to cells is immediately translated and cannot be spatially controlled.

We recently developed photocaged 5′ cap analogues (FlashCaps) that can be incorporated into any given mRNA by *in vitro* transcription.^13^ The resulting mRNAs are translationally muted until being irradiated. Light removes the single PPG and liberates the free cap0 structure, allowing translation to initiate. For proof-of-principle, we already demonstrated the activation of translation for a number of reporter-mRNAs *in vitro*, in cells and in zebrafish, but not for a protein with a functional effect on cells.^13–17^

We reasoned that gene editing with Cas9 and guide RNAs could enormously benefit from a light-mediated mRNA activation approach, as it would allow temporal and spatial control of gene editing. The intrinsically short expression time starting from mRNA, which can be a limitation for other functional proteins, would be an advantage in this case, as it prevents off-targeting.

Here, we report light-controlled gene editing based on a translationally muted Cas9-mRNA. The approach builds on previously developed FlashCaps and their incorporation into mRNA by *in vitro* transcription. We show that Cas9 protein is readily produced in HEK293T cells after irradiation of the photocaged mRNA precursor. In contrast to the previously tested reporter-mRNAs, it was important to optimize the 5′ UTR for cap-dependent translation to accomplish efficient light-mediated knock-out in cells.

## Results and discussion

**Fig. 1:**
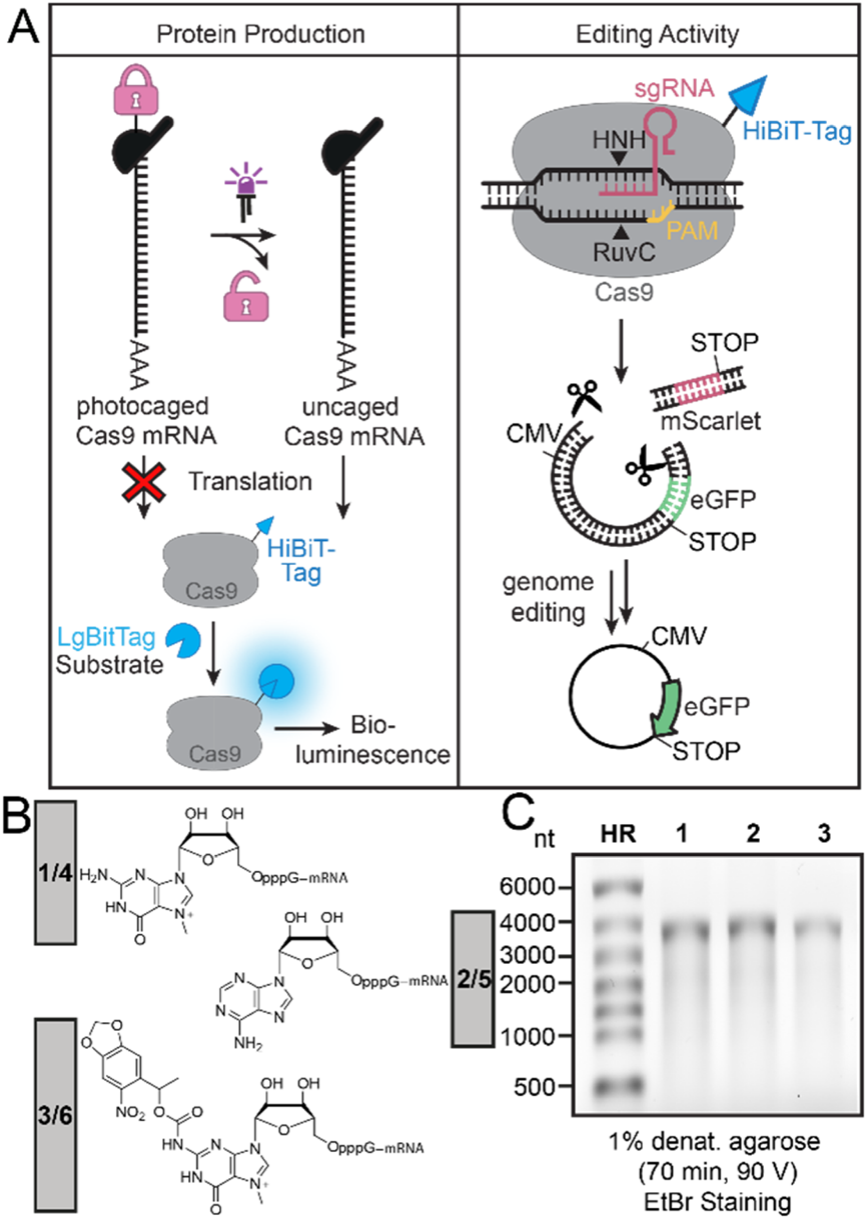
(A) Concept of photo-activation of Cas9-mRNA translation to activate genome editing. (Left panel) The light-activated production of Cas9 protein is directly evaluated *via* the HiBit-tag and bioluminescence. (Right panel) The light-activated gene editing activity is studied using a dual fluorescence reporter system. Active Cas9 removes the red reporter and successful editing therefore increases the ratio of eGFP/mScarlet fluorescence. This fluorescence change after gene editing in cells is evaluated by flow cytometry, Western blot and fluorescence microscopy. (B) Different 5′ caps on Cas9-mRNA with original (1, 2, 3) and optimized RpS25 (4, 5, 6) 5′ UTR used in this study. Cap0 (*i.e.* m^7^GpppG) (1 or 4), ApppG (2 or 5) and the synthesized NPM-FlashCap (3 or 6). (C) Denaturing agarose gel of differently capped Cas9-mRNAs (1, 2, 3). HR: RiboRuler High Range marker.

To achieve light-controlled genome editing by activation of Cas9-mRNA translation, we wanted to generate a Cas9-mRNA with a PPG at the 5′ cap to block translation (Fig. 1A). Irradiation should release Cas9-mRNA with a cap0 structure and thus initiate production of Cas9 protein in cells, which can be quantified by a functional tag.

Here, we use the HiBiT-tag to quantify the amount of Cas9 protein production. The assay works by addition of the LgBiT to reconstitute a functional luciferase (termed NanoBiT), and the luciferase substrate. Conversion of the substrate produces a bioluminescence signal that is proportional to the amount of protein (Fig. 1A, left panel). The most important functional proof will be gene editing activity achieved by the resulting Cas9 protein. The gene editing activity can be assessed in a quantitative way by a dual reporter (Fig. 1A, right panel). Two sgRNAs targeting the region upstream and downstream of the mScarlet coding sequence were used to guide mScarlet cleavage and activate eGFP gene expression. The dual reporter assay and measurement of eGFP/mScarlet fluorescence permits quantification of the editing activity.

To make a translationally muted Cas9-mRNA, we cloned a construct containing the Cas9 gene with a C-terminal HiBiT-tag in the pmRNA vector (ESI Fig. 1†) and produced the ∼4000 nt long mRNA by *in vitro* transcription using T7 RNA polymerase and different 5′ caps for transcriptional priming (Fig. 1B). Specifically, we used m^7^GpppG to generate cap0-Cas9-mRNA (1) as positive control and ApppG to generate the negative control ApppG-Cas9-mRNA (2), which does not show cap-dependent translation. To achieve light-dependent activation of translation, we used the previously reported NPM-FlashCap,^13^ carrying a nitropiperonylmethyl (NPM) group at the N^2^ position of the m^7^G of the 5′ cap to generate FlashCap-caged-Cas9-mRNA (3). All 5′ cap structures and resulting mRNAs are shown in Fig. 1B and C. To obtain exclusively capped Cas9-mRNA after the IVT, uncapped mRNA was routinely digested using polyphosphatase and Xrn1.

### Light-activated Cas9 protein production in cells

With photocaged-Cas9-mRNA (3) in hand, we tested the light-mediated production of Cas9 protein in cells. HEK293T cells were transfected with the differently capped Cas9-mRNAs (1–3). At 4 h post transfection, cells were either briefly irradiated (365 nm, 10 s) or kept in the dark (controls) and then incubated until 48 h post transfection.

**Fig. 2:**
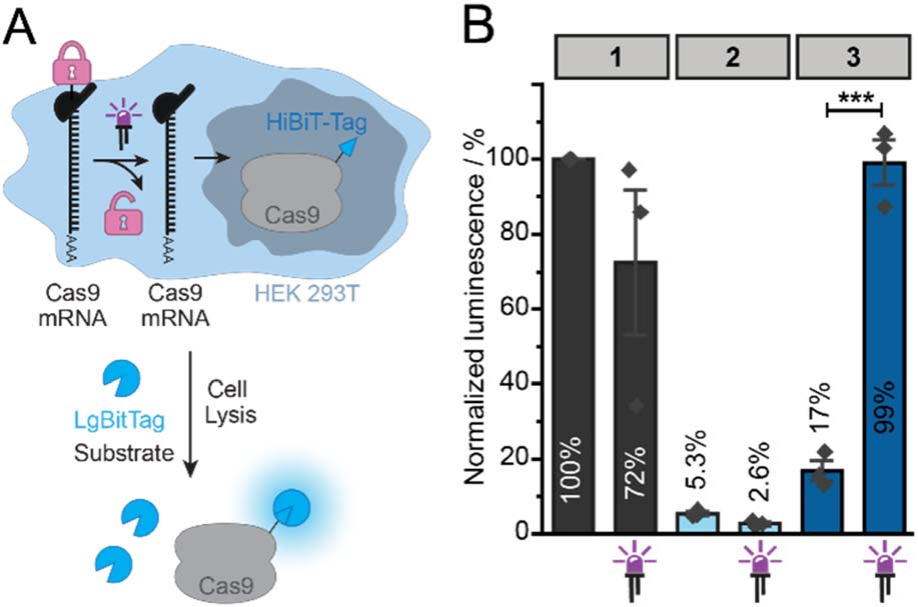
Light-mediated activation of Cas9-mRNA translation. (A) HiBiT assay to measure Cas9 protein production from differently capped Cas9-mRNAs. (B) Translation of differently capped Cas9-mRNAs (1, 2, 3) in HEK293T cells. The amount of protein produced is determined *via* the HiBiT-tag. Luminescence signals normalized to unirradiated cells transfected with cap0-Cas9-mRNA. LED icons indicate irradiated samples (365 nm, 10 s). Bars and error bars show mean value ±SEM of *n* = 3 independent experiments. Statistical significance was determined by two-tailed Student's *t*-test. Significance levels were defined as *P < 0.05, **P < 0.01, ***P < 0.001.

The amount of Cas9 protein produced was then analyzed *via* the 11 amino acid long HiBiT-tag at the C-terminus using the Nano-Glo® HiBiT Lytic Detection System (Fig. 2A).^18^ For this purpose, the cells were harvested by adding a lysis mix containing polypeptide LgBiT and the substrate furimazine. The LgBiT peptide interacts with the HiBiT-tag to form the NanoBiT enzyme that converts the substrate to release a bioluminescence signal. This bioluminescence signal is proportional to the amount of HiBiT-tagged protein.

Quantification of the bioluminescence showed the highest signal for cap0-Cas9-mRNA (1), which was used for normalization (100%) (Fig. 2). Irradiation reduced the signal of the positive control 1 to 70% (Fig. 2B). The reduction of translation as a result of UV-irradiation is known and has been observed before for reporter-mRNAs (FlashCaps).^13^ The photocaged-Cas9-mRNA (3) was muted, showing only 17% of signal with respect to the positive control (1), indicating that the PPG at the 5′ cap efficiently reduces translation of the Cas9-mRNA. After irradiation of cells transfected with 3, a very strong signal was measured, *i.e.* 99% compared to the positive control, indicating that brief irradiation at 365 nm successfully uncages the photocaged-Cas9-mRNA (3) to form functional cap0-Cas9-mRNA (1), which is translated efficiently to the respective protein. The negative control (ApppG-Cas9-mRNA (2)) showed very little background signal (5%), indicating that the Cas9-mRNA construct is subject to only little cap-independent translation (Fig. 2B).

From these data, the amount of cap-dependent translation of 3 can be obtained by subtracting the background signal of 2 from all values (ESI Table 4†). Cap-dependent translation of 3 was calculated to be 12% before irradiation and 99% after irradiation. Taken together, the light-mediated activation of NPM-Cas9-mRNA (3) is 6-fold in general and 8-fold for cap-dependent translation for this construct and conditions. As the PPG at the 5′ cap can only interfere with cap-dependent translation but not cap-independent initiation by the ribosome, this calculation is relevant. However, the background translation is also important to notice (even if cap-independent) and reveals that the NPM-Cas9-mRNA – although muted – shows leakiness of translation.

### Development of dual fluorescence reporter system

**Fig. 3:**
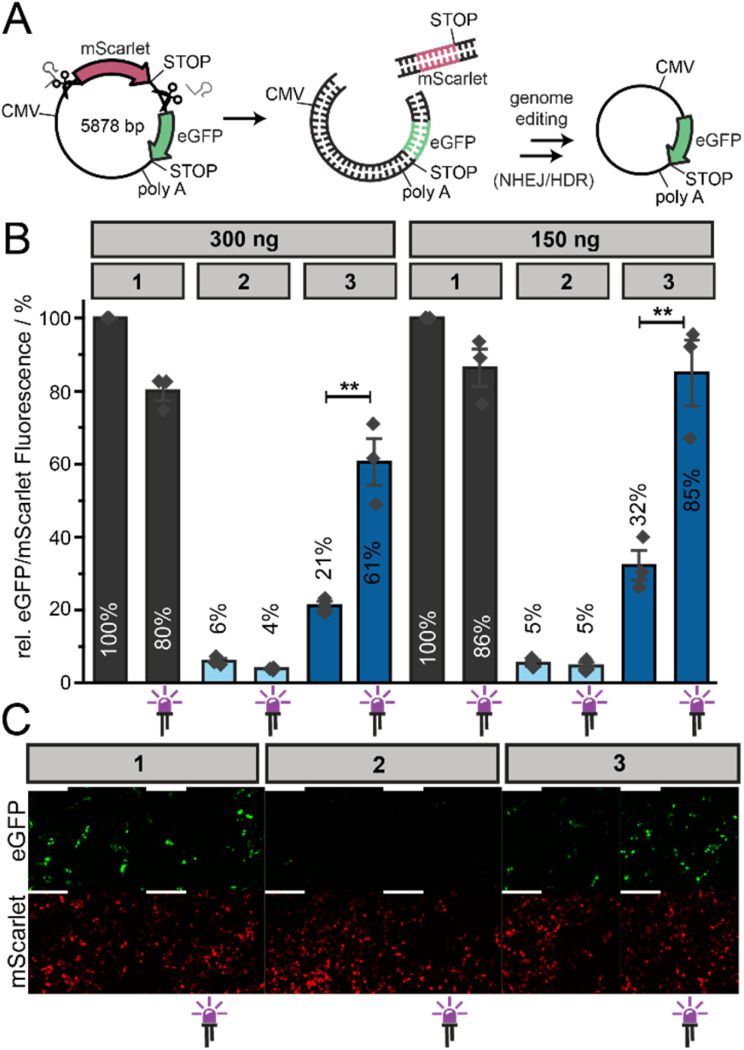
Light-activated gene editing *via* photocaged Cas9-mRNA in mammalian cells. (A) Concept of the Cas9-mediated excision of the mScarlet ORF in the dual fluorescence reporter system, activating eGFP gene expression. (B) Relative eGFP/mScarlet fluorescence signal measured by flow cytometry. HEK293T cells were first transfected with different amounts (300 ng, 150 ng) of differently capped Cas9-mRNAs (1: cap0, 2: ApppG, 3: NPM-FlashCap) and the two sgRNAs. Cells were left in the dark or irradiated after 4 h (LED: 365 nm, 10 s). After additional 4 h, cells were transfected with the dual fluorescence reporter system. Bars and error bars show mean value ±SEM of *n* = 3 independent experiments. Statistical significance was determined by two-tailed Student's *t*-test. Significance levels were defined as *P < 0.05, **P < 0.01, ***P < 0.001. (C) Microscopy images of fixed HEK293T cells that were transfected as in B with 150 ng Cas9-mRNA. The scale bar is 200 μm.

Next, we wanted to test, whether activation of Cas9-mRNA translation will trigger gene editing by light. To get quantitative insights into Cas9 activity, we used a dual reporter system, in which eGFP expression becomes activated after successful Cas9 cleavage (Fig. 1A, right panel). To this end, we cloned mScarlet downstream of the CMV promoter and upstream of the eGFP coding sequence (Fig. 3A). In this dual reporter, a stop codon downstream of mScarlet prevents eGFP expression.

We designed two sgRNAs aiming to remove the mScarlet gene by Cas9 and allow eGFP expression (Fig. 3A). One sgRNA binds upstream of the mScarlet coding sequence and the other one downstream of the stop codon (Fig. 3A). These sgRNAs were produced by *in vitro* transcription. We validated the functionality of the dual reporter in combination with the designed sgRNAs by an *in vitro* cleavage assay (ESI Fig. 2†), using recombinantly produced Cas9 protein. Gel electrophoresis confirmed that the sgRNAs led to specific and efficient cleavage of the dual reporter DNA *in vitro* (ESI Fig. 2†). Based on the design and the *in vitro* results the dual reporter should work. Cas9-mediated cleavage should remove the mScarlet gene from the reporter and ultimately result in eGFP expression. The ratio of eGFP/mScarlet fluorescence can then be used to assess the efficiency of genome editing.

### Light-activated Cas9 activity

Next, we sought to implement the dual reporter in cells and test whether Cas9-based editing can be achieved as a result of light-mediated activation of Cas9-mRNA translation. To achieve this, HEK293T cells were transfected with the differently capped Cas9-mRNAs and the corresponding sgRNAs targeting the dual fluorescence reporter system. Following a 4 h incubation period, cells were either irradiated (365 nm, 10 s) or kept in the dark. Four hours later, the media was exchanged, and the cells were transfected with the dual fluorescence reporter plasmid. At 48 h post-transfection, HEK293T cells were harvested for flow cytometry (ESI Fig. 3B†) or fixed for microscopy analysis (Fig. 3C).

The flow cytometric analysis for eGFP and mScarlet showed a clear difference for the positive (1) and negative (2) control Cas9-mRNAs (ESI Fig. 3†), indicating that the dual reporter functions in cells and that gene editing is affected by the efficiency of translation of Cas9-mRNA. For quantification, the eGFP/mScarlet fluorescence obtained after transfection of the positive control, *i.e.* cap0-Cas9-mRNA (1) was again set to 100% (Fig. 3B). Irradiation of cells transfected with 1, slightly reduced this ratio (Fig. 3B), in line with the previous observation that UV irradiation has negative impact on translation and Cas9 protein formation (Fig. 2). Transfection of cells with the negative control (2) gave a very low eGFP/mScarlet ratio (Fig. 3B), confirming that the genome editing of the dual reporter starting from Cas9-mRNA depends on its translation and its 5′ cap structure.

When we tested the photocaged-Cas9-mRNA (3), flow cytometric analysis showed a very low ratio, *i.e.* 21% compared to the positive control (1) (if 300 ng mRNA were transfected). After irradiation, this value is increased to 61%, which comes close to the irradiated positive control (∼80%) (Fig. 3B). These data are the first proof-of-principle that gene editing can be achieved by light-mediated activation of Cas9-mRNA translation. However, the background activity without irradiation is quite high and, as a consequence, the observed increase is rather moderate.

Aiming to mitigate background editing, we reduced the amount of Cas9-mRNA. However, halving the amount of Cas9-mRNA used for transfection did not lower the background activity for photocaged-Cas9-mRNA relative to the positive control (Fig. 3B). For 150 ng NPM-Cas9-mRNA (3) we observed 32% relative eGFP/mScarlet signal before and 85% after irradiation (Fig. 3B and ESI Fig. 3†).

When we analyzed the cells by confocal microscopy, the same trend was observed (Fig. 3C). The positive control (1) yielded the strongest eGFP fluorescence and irradiation slightly decreased the editing. The negative control (2) showed only very little eGFP fluorescence, indicating little to no editing. The photocaged-Cas9-mRNA (3) showed a clear light-dependent increase in editing (Fig. 3C). However, there was considerable eGFP fluorescence background in the absence of light, indicating that the leakiness of the photocaged-Cas9-mRNA (evident from Fig. 2) is of relevance for the observed gene editing background. The data confirm the results obtained by flow cytometry and suggest that small amounts of Cas9 protein produced can have noticeable effects on editing.

### Optimization of light-mediated genome editing *via* photocaged Cas9-mRNA

As reducing the amount of mRNA did not decrease the background editing observed with photocaged Cas9-mRNA, we looked for alternative solutions. Aiming to increase the light-mediated activation of gene editing by photocaged Cas9-mRNA, we hypothesized that optimizing Cas9-mRNA towards more cap-dependent (*versus* cap-independent) translation could be a viable option to reduce the leakiness and improve the light-mediated activation. The 5′ UTR is pivotal for ribosome recruitment.^19^ Highly structured motifs in the 5′ UTR – such as pseudoknots, hairpins – but also upstream open reading frames (uORFs) can inhibit cap-dependent translation.^20^ Given the significant impact of the 5′ UTR on translation efficiency,^20^ we decided to investigate whether variations in the 5′ UTR could enhance light-activated cap-dependent Cas9-mRNA translation.

**Fig. 4:**
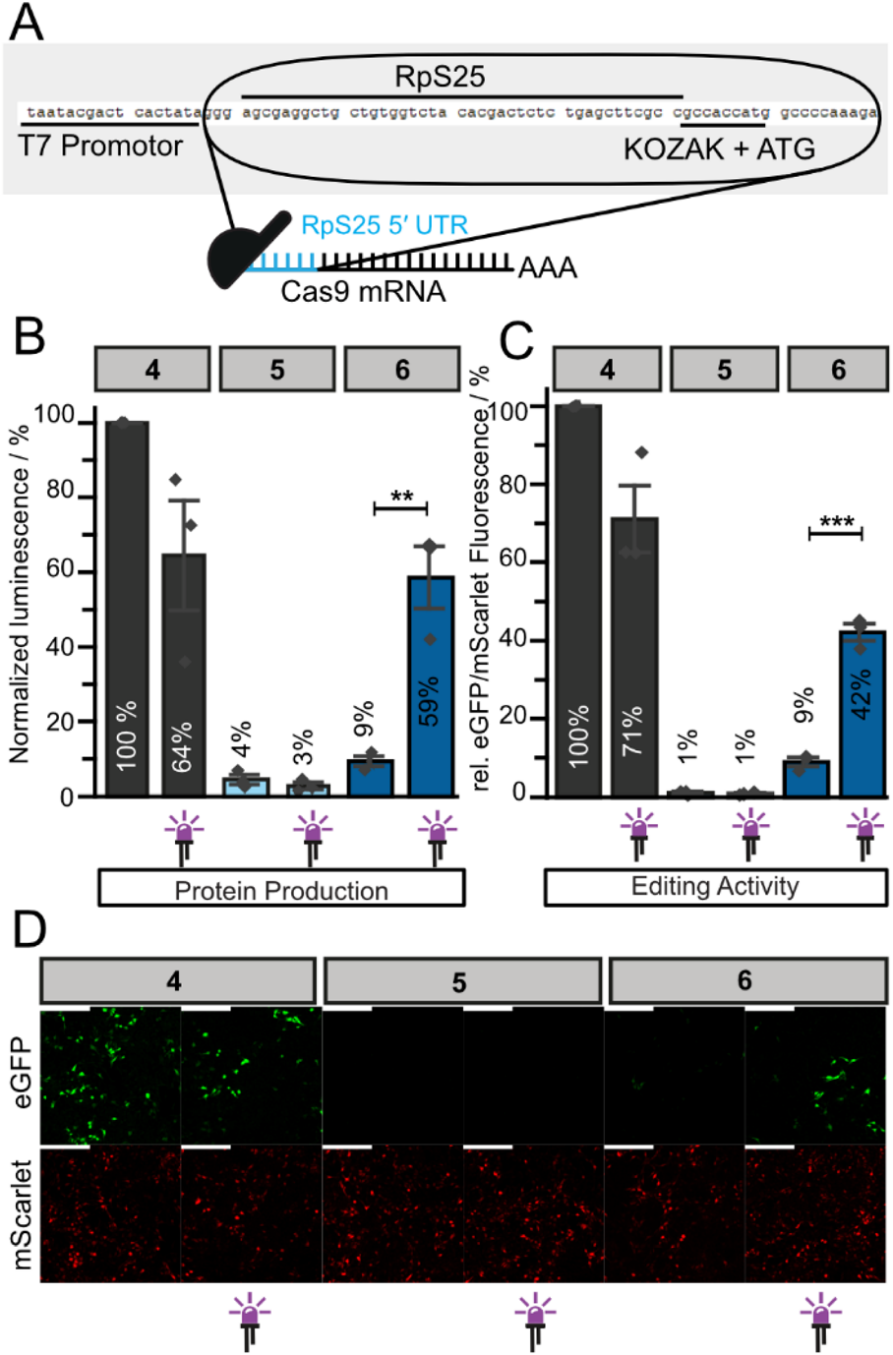
Light-activated Cas9 protein production and protein function with an optimized Cas9-mRNA. (A) Optimized RpS25-Cas9-mRNA (below) produced by IVT of the DNA template (on top). (B) Normalized luminescence signal of differently capped Cas9 protein using the HiBiT-tag. (C) Relative eGFP/mScarlet fluorescence signal measured by flow cytometry. HEK293T cells were first transfected with differently capped Cas9-mRNAs (4: cap0, 5: ApppG, 6: NPM-FlashCap, 500 ng) and the two sgRNAs. Cells were left in the dark or irradiated after 4 h (violet LED: 365 nm, 10 s). After additional 4 h, cells were transfected with the dual-fluorescence reporter system. Bars and error bars show mean value ± standard mean of error (SEM) of *n* = 3 independent experiments. Statistical significance was determined by two-tailed Student's *t*-test. Significance levels were defined as *P < 0.05, **P < 0.01, ***P < 0.001. (D) Microscopy images of fixed HEK293T cells that were transfected as in B. The scale bar is 200 μm. Higher resolution images in ESI Fig. 11.†

The originally used Cas9-mRNA construct (ESI Fig. 5†) had a very short 5′ UTR (5 nt to first AUG) and lacked a Kozak sequence. Furthermore, it contained a second AUG codon directly upstream of the nuclear localization signal (NLS) of the Cas9 open reading frame (ORF). First, we tried to improve the mRNA construct by inserting the Kozak sequence and using the AUG further downstream, while simultaneously removing the FLAG tag (ESI Fig. 4A†). However, in this construct the overall editing activity remained very low. A recent study by Leppek *et al.* investigated the influence of the 5′ UTRs on translation.^21^ Following their insights and looking for relatively short 5′ UTR for practical reasons, we chose the 5′ UTR from human hemoglobin subunit beta (hHBB) and ribosomal protein RpS25, which had shown higher expression than hHBB. This 50 nt or 41 nt long sequence was inserted between the T7 promoter and the KOZAK sequence (Fig. 4A and ESI Fig. 4C†). The new constructs were termed hHBB- and RpS25-Cas9-mRNA and produced by IVT with different 5′ caps, in analogy to the original construct (ESI Fig. 5†).

Next, we investigated how the RpS25 5′ UTR influences the activation of cap-dependent translation of the photocaged mRNA. Using the HiBiT assay (Fig. 2A), we tested whether the 5′ UTR of RpS25 affected the light-mediated activation of translation by testing differently capped RpS25-Cas9-mRNAs (4–6). The cap0-RpS25-Cas9-mRNA (4) was set to 100% and irradiation (365 nm, 10 s) reduced the amount of protein produced (64%, Fig. 4B), in line with the results for the original constructs (Fig. 2B). The negative control (ApppG-RpS25-Cas9-mRNA, 5) gave a very low signal (4%). To our delight, the photocaged-RpS25-Cas9-mRNA (6) caused a low signal (9%, Fig. 4B), lower than the original photocaged construct 3 (17%, Fig. 2B). After irradiation, the signal was increased to 59%, almost reaching the level of the irradiated positive control (6, 64%, Fig. 4B). Calculating the amount of cap-dependent activation by subtracting the cap-independent translation (4%), the new photocaged construct NPM-RpS25-Cas9-mRNA (6) can be activated 11-fold and therefore performs better than the original construct 3 (Fig. 2B, 8-fold). More importantly, the new construct seems to be less leaky, as the background signal is reduced from 17% (Fig. 2B) to 9% (Fig. 4B).

With these promising activation properties, we turned our attention again to the light-activated gene editing (Fig. 3A). Using the dual reporter in combination with the RpS25-Cas9-mRNA construct, we analyzed the editing activity again by flow cytometry (Fig. 4C) and fluorescence microscopy (Fig. 4D). In flow cytometry, the fluorescence ratio was normalized to the positive control (cap0-RpS25-Cas9-mRNA, 4, Fig. 4C). Again, we saw a slight reduction in editing activity of 4 upon irradiation (Fig. 4C). The negative control 5 showed very little editing (1%) (Fig. 4C), lower than the original construct in the same assay (5–6%, Fig. 3B). This reduction suggests that, as intended, the 5′ UTR of RpS25 indeed causes very little cap-independent translation. The photocaged RpS25-Cas9-mRNA (6) showed only 9% of editing activity compared to 1 (Fig. 4C). This is much lower than for the original construct (32%, Fig. 3B). After irradiation, the editing activity by 6 was increased to 42%, which is a 5.2-fold increase (Fig. 4C). The original construct had yielded a 3-fold activation of gene editing. The same set of experiments was conducted with the hHBB 5′ UTR, but this construct performed worse than the original one and was not further pursued (ESI Fig. 4D†). Although the absolute amount of editing achieved with Cas9-mRNA 3 after photoactivation was higher in the original construct (61% *versus* 42%), the RpS25-Cas9-mRNA construct 6 is better suited for light-activated gene editing due to the lower background and higher light-mediated activation.

**Fig. 5:**
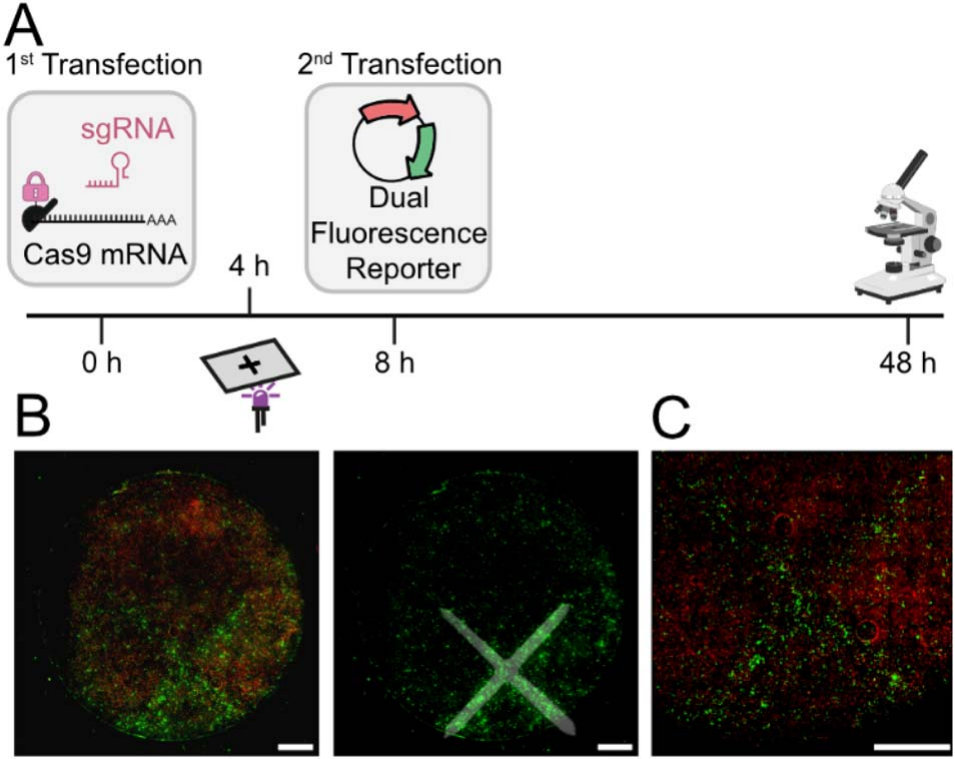
Local activation of gene editing by photoactivation of Cas9-mRNA. (A) Experimental scheme. Photomask was placed on the outside of the well. HEK293T cells were transfected with the mRNA (500 ng HiBiT-tagged FlashCap-Cas9-mRNA with RpS25 5′ UTR) and sgRNAs. At 4 h post-transfection, cells were irradiated through a photomask with a cross (violet LED: 365 nm, 30 s). At 8 h post-transfection, cells were transfected with the dual fluorescence reporter. (B) Fluorescence microscopy images of fixed HEK293T cells, showing overlay of eGFP and mScarlet (left) or eGFP channel with position of photomask indicated (right). (C) Higher magnification scan of the cells in B. Scale bar is 2 mm. Complementary images taken by a Typhoon imager are shown in ESI Fig. 12.†

Evaluation by fluorescence microscopy and Western blots confirmed these results for the differently capped Cas9-mRNAs (Fig. 4D and ESI Fig. 8†). Most importantly, cells transfected with photocaged RpS25-Cas9-mRNA (6) show very little eGFP signal (Fig. 4D). After irradiation of cells transfected with 6, Cas9-mediated gene editing is activated and eGFP is evident (Fig. 4D). Direct comparison of the results for the original (Fig. 3C) and 5′ UTR of RpS25 (Fig. 4D) constructs reveals that the new construct is improved. The efficient light-activated editing is clearly visible from the differences in eGFP fluorescence in the presence/absence of prior irradiation.

In addition, we independently validated the light-mediated gene editing activity by sequencing (ESI Fig. 7†). Comparing sequencing from cells transfected with photocaged RpS25-Cas9-mRNA (6) and with or without irradiation and controls (4, 5) confirmed that irradiation increased gene editing by 6.

As the photocaged RpS25-Cas9-mRNA gave promising results, we were curious to assess whether local irradiation could be used to exert spatial control of gene editing. In a proof-of-concept, we used a photomask in the shape of a cross with bars of approximately 350 μm width and performed the editing experiment with the dual reporter. Irradiation *via* the cross resulted in green fluorescence with this shape, indicating that only this subset of cells had received light and become irradiated (Fig. 5B and C). Imaging in a Typhoon imager confirmed the results for the cross-shaped and a slit-shaped photomask (ESI Fig. 12†). These data show that light can be used to activate gene editing starting from a photocaged Cas9-mRNA with temporal and spatial control.

## Conclusions

In conclusion, we demonstrated optical activation of Cas9 protein production and Cas9-mediated gene editing from a translationally muted Cas9-mRNA. In contrast to previous work with reporter proteins whose function scales linearly over a broad range, it was important to reduce the inherent leakiness of the system before uncaging. The optimized 5′ UTR of the Rps25-Cas9-mRNA construct reduced background activity. As a result, the light-mediated activation of Cas9-mRNA translation improved to 11-fold and the light-mediated gene editing was augmented to 5-fold. Gene editing starting from Cas9-mRNA already benefits from limited half-life of mRNA. Together with the light-mediated activation of translation, it allows to start editing at chosen timepoints and locations.

## Author contributions

A. R. conceived and supervised the study. H. S., G. C. D. and M. S. designed and performed all experiments. S. H. performed cell culture experiments. All authors discussed the results. A. R. and H. S. wrote the manuscript. H. S., G. C. D., M. S., and A. R. revised the manuscript.

## Conflicts of interest

N. K., F. W., and A. R. are the inventors on a European patent application (EP1184349.5, pending) of the University of Münster covering the synthesis and use of photo-cleavable 5′ cap analogues as well as RNA molecules comprising photo-cleavable 5′cap analogues.

## Supplementary Material

SC-016-D5SC01999K-s001

## Data Availability

Data supporting this manuscript can be found in the ESI.†
