# Supplementary material for: Light-controlled genome editing by activation of Cas9-mRNA translation
Source: Chem Sci. 2025 Jun 30;16(30):13916–22. doi: 10.1039/d5sc01999k (PMC12230757; doi:10.1039/d5sc01999k)
Supplement: SC-016-D5SC01999K-s001 [file SC-016-D5SC01999K-s001.pdf]

Supplementary Information (SI) for Chemical Science.
This journal is © The Royal Society of Chemistry 2025

Material and Methods

FlashCaps were synthesized and evaluated as previously described.[1]

Plasmids

The pRNA2-(A)128 was a gift from Stephen Ikeda (Addgene plasmid # 174006 ; http://n2t.net/addgene:174006 ; RRID:Addgene_174006).[2] The pMJ806 (pET-Cas9) was a gift from Jennifer Doudna (Addgene plasmid #39312; http://n2t.net/addgene:39312; RRID:Addgene_39312).[3] pMSCV-U6sgRNA(BbsI)-PGKpuro2ABFP was a gift from Sarah Teichmann (Addgene plasmid # 102796 ; http://n2t.net/addgene:102796 ; RRID:Addgene_102796).[4] The bbCas9pluspAAA was a gift from Ivo Huijbers (Addgene plasmid # 82581 ; http://n2t.net/addgene:82581 ; RRID:Addgene_82581).[5]

Primer

Supplementary Table 1: Used DNA oligos throughout this work.

Name	Sequence 5′ → 3′	Description
fwd_XhoI_mScarlett	ATCTCGAGGCCACCATGAGTAAAGGAGAAG	Generation of Dual Fluorescence Reporter System
rev_EcoRI_mScarlett	ATGAATTCTTTATTCGCGAGCTGCGGCCGCTTTATTTG	Generation of Dual Fluorescence Reporter System
fwd pMSCV mScarlett_start	CACCGATCACTAGACGCTTTATTGGT	Cloning of sgRNA
rev pMSCV mScarlett_start	TAAAACCAATAAAGCGTCTAGTGATC	Cloning of sgRNA
fwd pMSCV T7 mScarlett_start	TTAATACGACTCACTATAGATCACTAGACGCTTTATTG	Generation of DNA Template for IVT
fwd pMSCV mScarlett_end	CACCGTTCTGCAGTCGACGGTACCGGT	Cloning of sgRNA
rev pMSCV mScarlett_end	TAAAACCGGTACCGTCGACTGCAGAAC	Cloning of sgRNA
fwd pMSCV T7 mScarlett_end	TTAATACGACTCACTATAGTTCTGCAGTCGACGGTACCG	Generation of DNA Template for IVT
Standard-rev T7 sgRNA	AAAAGCACCGACTCGGTGCC	Generation of DNA Template for IVT
Seq Cas9 before T7	TGTGATGCTCGTCAGGGGGG	Sequencing Primer
Seq Cas9 1	CAAGCTGTTCATCCAGCTGG	Sequencing Primer
Seq Cas9 1.5	TGGAACTTCGAGGAAGTGG	Sequencing Primer
Seq Cas9 2	GAGGACTACTTCAAGAAAAT	Sequencing Primer
Seq Cas9 3	GACAACAAGGTGCTGACCAG	Sequencing Primer
Seq Cas9 4	GACTGGGACCCTAAGAAGTA	Sequencing Primer
Seq Cas9 5	AGAGTGATCCTGGCCGACGC	Sequencing Primer
Fwd HIBIT bbCas9	GTGAGCGGCTGGCGGCTGTTCAAGAAGATTAGCTAAGAATTCCTAGAGCTCGCTAA	Insertion of the C-terminal HiBiT-Tag
Rev HIBIT bbCas9	CTTTTTCTTTTTTGCCTGGCCGGCCTTTTTCGTGGCC	Insertion of the C-terminal HiBiT-Tag
Sequence RpS25 UTR	AGCGAGGCTGCTGTGGTCTACACGACTCTCTGAGCTTCGCC	

Fwd 5′ UTR RpS25	AGCGAGGCTGCTGTGGTCTACACGACTCTCTGAGCTTCGCCGCCACCATGGCCCCAAAGAAGAAGCGGAAGGTC	Cloning of RpS25 5′ UTR
Rev 5′ UTR bbCas9	CCCTATAGTGAGTCGTATTATCTAGATGGGTCCTGCAG	Cloning of RpS25 5′ UTR
Fwd_Dual fluorescence reporter	GGATAGCGGTTTGACTCACG	Target gene PCR amplification
Rev_Dual fluorescence reporter	GCGGACTTGAAGAAGTCGTG	Target gene PCR amplification
Seq Primer fwd	CAATGGGAGTTTGTTTTGGC	Sequencing Primer

Cloning to generate the Dual Fluorescence Reporter Plasmid

The mScarlet insert gene was PCR-amplified using the corresponding primers (fwd_XhoI_mScarlett and rev_EcoRI_mScarlett), dNTP mix (200 µM), Phusion High-Fidelity polymerase (0.02 U) in 1 × HF buffer (Thermo Scientific) in a total volume of 50 µL.

The pRNA2-(A)128 vector (6 µg) and the insert were restricted using XhoI and EcoRI (Thermo Scientific) for 30 min at 37 °C, followed by inactivation for 5 min at 80 °C. To prevent recircularization of the pRNA2-(A)128 plasmid, FastAP alkaline phosphatase (6 U) was added and incubated at 37 °C for 15 min, followed by inactivation at 75 °C for 5 min. The plasmid DNA fragment was gel-purified (1% agarose, 1 h, 90 V) and isolated, whereas the restricted PCR template was purified using NucleoSpin Gel and PCR Clean-up kit (Macherey-Nagel) according to the manufacturer’s instructions.

Plasmid DNA (50 ng) and PCR template (5:1 molar ratio) were ligated by T4 DNA ligase (2.5 U, Thermo Scientific) in 1 × T4 buffer in a total volume of 5 µL. The ligation mixture was incubated for 16 h at 16 °C prior to inactivation for 10 min at 65 °C. 50–100 ng was used for transformation of electrocompetent *E. coli TOP 10* cells. The success of cloning was confirmed by Sanger Sequencing.

Oligo Design of sgRNA sequence for cloning into the sgRNA backbone vector and sgRNA IVT

The sgRNAs were cloned and prepared according to a protocol by Pritchard et al.[5] Suitable sgRNA target sites including the NGG PAM sequence were selected using the CRISPOR following website: http://crispor.tefor.net/crispor.py. It classifies potential sgRNA sequences according to specificity and off-target sites.

For oligo design, the PAM sequence was removed and the first 5′ base was defined to start with a G. For sticky-end ligation, the sequence 5′-CACC-3′ for the pMSCV vector backbone was added to the forward primer. The reverse primer starts with 5′-TAAAAC-3′ followed by the reverse complement sequence of the sgRNA. Thus, forward and reverse primer were designed

to include the appropriate overhang sequence. For preparation of the T7 DNA template, the primer was acquired by the addition of 5′-TTAATACGACTCACTATAG-3′ upstream the sgRNA sequence. The standard reverse primer is 5′-AAAAGCACCGACTCGGTGCC-3′. Primers were purchased from Biolegio and dissolved to 100 µM in ddH_2O.

The sgRNA backbone vector (pMSCV-U6sgRNA(BbsI)-PGKpuro2ABFP) (6 µg) was incubated with FastDigest BpiI (Thermo Scientific) restriction enzyme at 37 °C for 30 min, followed by inactivation at 65 °C for 10 min. The vector backbone was gel purified on 1% agarose gel and purified via NucleoSpin Gel and PCR Clean-up kit (Macherey-Nagel).

9 µL of the forward and 9 µL of the reverse primer in 2 µL 10 × NEBuffer 3.1 were annealed (98 °C for 1 min, 48 °C for 30 min and stored at 4 °C) to provide the insert with the appropriate overhang sequence. The annealed insert was diluted 1:625 and 1 µL was used for ligation reaction with the restricted sgRNA backbone vector.

For PCR amplification, sgRNA plasmids (70 ng) was mixed with dNTPs (200 µM), forward and reverse T7 oligos (100 µM) and Phusion High-Fidelity polymerase (0.02 U) in 1 × HF buffer (Thermo Scientific) in a total volume of 50 µL. The following PCR program (Supplementary Table 2) was used to obtain the DNA template of approximately 120 bp containing the T7 promoter. DNA integrity was checked on 3% agarose gel.

Supplementary Table 2: Temperature program for generation of DNA template for sgRNA.

Phase	Temperature	Time	Cycle
Initial Denaturing	98 °C	2 min	1
Denaturing	98 °C	4 s	22
Annealing	55 °C	5 s	
Elongation	72 °C	1 s	
Final Elongation	72 °C	1 min	1

Protein production of Cas9

The pET-Cas9 plasmid was used for Cas9 protein production, which was produced from an adapted protocol of Jinek et al.[3] Under semi-sterile conditions, the main culture was inoculated with 1/100 (v/v) preculture in 2YT medium and incubated at 37 °C and 180 rpm. After bacteria were cultured to an optical density of OD_{600} = 0.6 at λ = 600 nm, protein production was induced by the addition of 0.2 mM Isopropyl β-D-1 thiogalactopyranoside (IPTG). The main culture was incubated at 18 °C for 16 h. Then, cells were harvested by centrifugation for 30 min, 5000 × g at 4 °C. Cell pellets were resuspended, combined and centrifuged again for 20 min, 4000 rpm at 4 °C. Cell pellets were frozen in liquid nitrogen and stored at -20 °C. The cell pellet was thawed on ice and resuspended in 3 $mL \cdot g^{-1}$ weight in lysis buffer (20 mM Tris, 500 mM NaCl,

supplemented with Complete protease inhibitor, pH 8). Cell lysis was done by sonication (5 min, 0.5 s pulse on, 0.5 s pulse off, 35% amplitude, on ice). Subsequently, cell debris was removed by centrifugation (30 min, 12,000 × g, 4 °C). The supernatant containing the soluble His-tagged protein was purified by NiNTA affinity chromatography on 1 mL HisTrapTM FF column with ÄKTApurifier chromatography system in wash buffer (20 mM Tris, 500 mM NaCl, 20 mM Imidazole). Protein was eluted from the column using elution buffer (20 mM Tris, 500 mM NaCl, 200 mM Imidazole) with the following buffer gradient (gradient: 0 - 14 min 0%, 14 - 19 min 10%, 28 min 100%), monitored by absorption at λ = 280 nm and checked for full-length protein on SDS PAGE. Fractions containing the desired protein were pooled and dialyzed overnight against storage buffer (20 mM HEPES, 150 mM KCl, 1 mM TCEP, 10% glycerol, pH 7.4). To remove the MBP tag, TEV protease was added. Ion exchange chromatography was performed on HiTrap S column using the Äktapurifier system. A linear gradient of 100 mM KCl to 1 M KCl was used to elute bound Cas9 protein (20 mM HEPES). To obtain RNAse-free proteins, an additional size exclusion chromatography step was added using GeFi Superdex 200 Increase 10/300 GL column (GE Healthcare) with gel filtration buffer (20 mM HEPES, 150 mM KCl, 1 mM TCEP). Fractions showing no RNase activity were combined and concentrated using Amicon Ultra-4 centrifugal filters (MWCO 100 kDa). Finally, the protein concentration was determined by comparison with BSA standards on 12% SDS PAA gels.

Seamless cloning of the RpS25 5′ UTR upstream of the Cas9 ORF

For seamless cloning without suitable restriction sites, the forward primer was designed with the insert as a 5′-overhang. The reverse primer hybridizes to the nucleotides upstream of the forward primer. The entire plasmid is amplified with both primers. The PCR is performed as described in Supplementary Table 3 with an extended elongation time sufficient to amplify the entire plasmid.

Supplementary Table 3: Temperature program for seamless cloning.

Phase	Temperature	Time	Cycle
Initial Denaturing	98 °C	30 s	1
Denaturing	98 °C	10 s	28
Annealing	72 °C	30 s	28
Elongation	72 °C	3 min	28
Final Elongation	72 °C	10 min	1

The DNA template was then digested by adding DpnI and incubating for 2 h. To generate the circularized plasmid, phosphorylation and ligation of the PCR product (50 ng) was performed by the addition of ATP (10 mM), T4 DNA polynucleotide kinase (5 U) and T4 DNA ligase (2.5 U)

in 10 × T4 ligase buffer in a reaction volume of 5 µL and incubation for 16 h at 16 °C. After transformation, positive colonies were identified by Sanger sequencing.

***In vitro* T7-transcription and purification of Cas9-mRNA**

The plasmid encoding Cas9 downstream of a T7 promotor was linearized using SapI (NEB). For this purpose, plasmid DNA (6 µg) was incubated with 1× FastDigest buffer or CutSmart Buffer and 6 µL of enzyme for 45 min at 37 °C, followed by inactivation at 65 °C for 20 min (SapI). By adding 6 µL of FastAP and incubation at 37 °C for 15 min, the ends were dephosphorylated. This was followed by inactivation at 65 °C for 5 min. The DNA T7 template was purified via NucleoSpin Gel and PCR Clean-up kit (Macherey-Nagel). The concentration was measured at 260 nm with NanoDrop 2000 (ThermoScientific) and linearization was proofed on 1% agarose gel. 600 ng of the linear dsDNA was used as template for T7 *in vitro* transcription (IVT) that was performed in the presence of A/C/UTP mix (0.5 mM), GTP (0.25 mM), the respective 5′ cap analogue (1 mM) (NPM-FlashCap, Ap_3G (Jena Bioscience) or m^7Gp_3G (Jena Bioscience)), T7 RNA polymerase (50 U; Thermo Scientific), RiboLock RNase Inhibitor (30 U; Thermo Scientific) and pyrophosphatase (0.1 U; Thermo Scientific) in 1 × transcription buffer (ThermoScientific) for 2 h at 37 °C. To remove remaining DNA template, DNase I (2 U; Thermo Scientific) was added and incubated for 1 h at 37 °C. The mRNA was purified using the RNA Clean & Concentrator-5 kit (Zymo Research). Uncapped RNA was removed by adding 10 U of RNA 5′-polyphosphatase (Epicentre) and incubation for 1 h at 37 °C. Subsequently, the 5′–3′ exoribonuclease XRN1 (NEB) and $MgCl_2$ (5 mM; Thermo Scientific) were added and the reaction mixture was incubated for 1 h at 37 °C. The capped mRNAs were purified using the RNA Clean & Concentrator-5 kit (Zymo Research). RNA integrity and length were confirmed by loading 100 ng mRNA onto 7.5% polyacrylamide (PAA) gel and electrophoresis was performed for 1.5 h at 12 W in 1 × Tris/Borate/ EDTA (TBE) buffer and imaged on Typhoon FLA 9500 scanner (GE Healthcare).

Transfection of HEK293T cells with Cas9-mRNA

One day prior to transfection, cells were seeded in 96-well plates (1.05 x 10^4 cells per well) in 150 µL or 12-well plates (7 x 10^4 cells per well) in 1 mL full medium. For microscopy experiments, cells were seeded on sterile 15 mm coverslips in 12-well culture plates. Due to the reduced adherence of HEK293T cells, coverslips were first moistened with poly-L-ornithine and incubated for at least 1 h at 37 °C before seeding the cells. Before transfection, cell media was exchanged to transfection medium omitting penicillin and streptomycin.

Transfection was performed by lipofection and varying mRNA amounts: 100 ng for 96-well plates, 150–300 ng of construct 1 and 500 ng of construct 2 for 12-well plates. For transfection,

the appropriate amount of mRNA was added to 5 µL Opti-MEM for 96-well format or 50 µL Opti-MEM for 12-well format. Moreover, 0.15 µL of Lipofectamine™ Messenger Max™ was combined with 5 µL of Opti-MEM for 96-well format or 1.5 µL of Lipofectamine™ Messenger Max™ was combined with 50 µL Opti-MEM for 12-well format, vortexed, and incubated for 10 min. Subsequently, the mRNA mixture is added to the liposome mixture, incubated for 5 min, and then added dropwise to the cells. Cells were incubated at 37 °C and kept in the dark or irradiated after 4 h.

Irradiation of HEK cells

Transfected HEK293T cells were irradiated in a 96-well plate at 365 nm (460 mW/cm^2) for 10 s using an LED with a collimator lens or 12-well plate without the collimator lens. The UV–A–LED (λ_{max} = 365 nm) was operated with 1300 mA. For spatial irradiation, a photomask in the shape of a cross with bars of approximately 350 µm width was used. Irradiation time was increased to 30 s. Media was here exchanged directly after irradiation to prevent diffusion of mRNA in the cell medium.

Transfection of HEK293T cells with plasmid DNA

At 8 h post transfection, cells were transfected with the dual fluorescence reporter plasmid in the dark. Transfection medium was exchanged with fresh transfection medium. 50 µL DMEM medium (Thermo Scientific) was mixed with the dual fluorescence reporter plasmid (300 ng) and 3 µL FuGENE® HD Transfection Reagent (Promega) and added to the cells after 20 min of incubation. Cells were incubated at 37 °C and kept in the dark.

HiBiT experiments

At 48 h post transfection, the protein production was assessed using the Nano-Glo® HiBiT Lytic Detection Kit (Promega). For this purpose, cell media was removed and 20 µL 1 × PBS were added. To this, 20 µL of Nano-Glo® HiBiT Lytic Reagent consisting of LgBiT protein and furimazine substrate in Nano-Glo HiBit Lytic Buffer was added according to the manufacturer's instruction. Cells were lysed on an orbital shaker at r.t. for 10 min, before the cells mixture was pipetted into white ELISA plates (Sarstedt). Luminescence intensity was measured on Infinite M1000pro microplate reader (Tecan)

Microscopy

At 48 h post transfection, cell media was removed and cells were fixed in 4% paraformaldehyde and incubated for 10 min at r.t. Cells were washed with 1 × PBS and the nucleus was stained by incubation with DAPI for 30 s. Cells were washed with 1 × PBS and ddH$_2$O and the coverslips were fixed on microscopy slides using Anti-Fade Fluorescence Mounting Medium (abcam). Fixed cells were analyzed on a Leica A TCS SP8 confocal laser scanning with a 10×

(air) or 20x (oil immersion) objective in eGFP and mScarlet channels (eGFP: λ_{Ex} = 488 nm, λ_{Em} = 510 nm, mScarlet: λ_{Ex} = 568 nm, λ_{Em} = 583–693 nm). Whole coverslip scans were carried out on a Leica DMi8 wide field fluorescence microscope equipped with a 5x (air) objective to identify the pattern transferred to the cells via illumination through the photomask. To enable a side-to-side comparison with the photomask, the fixed cells were additionally imaged on Typhoon FLA 9500 scanner (GE Healthcare) in the Cy2 channel.

Flow cytometry experiments

At 48 h post transfection, cells were harvested by adding 300 µL 1 × Trypsin/EDTA and incubation for 3 min. Subsequently, 600 µL HEK293T full media was added and the suspension was transferred into 1.5 mL Eppis. After centrifugation at 400 rpm for 5 min, cells were washed with 1 × PBS, centrifuged again and dissolved in 350 µL FACS buffer (1 × PBS, 25% FCS, 2 mM EDTA). Cell clumps were removed by pipetting through a 70 µm filter, fluorescence intensities were recorded by flow cytometry (SH800 Cell Sorter, Sony) on 130 µm chips. eGFP and mScarlet fluorescence were compensated and measured on FL2 525/60 (sensor gain 21%) and FL3 600/60 laser (sensor gain 24%), respectively. 100,000 cells (total cell count) were measured per sample. Analysis was performed with the Sony Analysis Software.

Western Blot

At 48 h post transfection, cell media was removed and cells were washed with 1 × PBS. Lysis was performed by incubating with CelLytic™ M (Sigma Aldrich) supplemented with 1 × cOmplete™ EDTA-free Protease Inhibitor Cocktail (Roche) for 15 min at r.t. Cell lysate was centrifuged at 21,300 × g for 15 min. The overall protein concentration was determined with ROTI®Quant (Carl Roth) according to the manufacturer's instruction. Absorbance was measured on Infinite M1000 pro microplate reader (Tecan). 30 to 40 µg of protein was separated using SDS page (10%, 120 V, 100 min). The proteins were transferred to the membrane (Amersham™ Protran™ 0.45 µm NC) using semi-dry blotting. From bottom to top, four layers of whatman® filter paper were combined with the membrane and the gel, followed by another four layers of whatman® filter paper. The stack was soaked in transfer buffer (3 g/L Tris, 14.4 g/L Glycin, 20% isopropanol) and blotted using V20-SDB (90 mA, 75 min, Scie-Plas). After blotting, membrane was washed once with 1 × PBS and incubated with BSA (3 % in 1 × PBS) for 1 h at r.t. The membrane was divided into two parts for incubation over night at 4 °C with either GFP antibody (B-2, Santa Cruz Biotechnology) or Nucleolin Monoclonal Antibody (Thermo Fisher Scientific). Afterwards, the membrane was washed thrice with Tween® 20

(0.01% in 1 × PBS) and once with 1 × PBS. Incubation with the polyclonal Rabbit Anti-Mouse Immunoglobulins/HRP antibody (Dako) was performed for 1 h at r.t., followed by washing as described before. For luminescence detection the SuperSignal™ West Pico PLUS chemiluminescence substrate (Thermo Fisher Scientific) was used according to the manufacturer's instructions and the membrane was imaged on Amersham™ Imager 680 (GE Healthcare). The relative signal was calculated as described below.

$$\text{Relative signal}_{\text{eGFP}} = \frac{\dfrac{\text{Signal}_{\text{Sample,eGFP}}}{\text{Signal}_{\text{Cap0 mRNA,eGFP}}}}{\dfrac{\text{Signal}_{\text{Sample,Nucleolin}}}{\text{Signal}_{\text{Cap0 mRNA,Nucleolin}}}}$$

In vitro stability

To test *in vitro* stability, 1.4 µg of differently capped Cas9-mRNA was incubated in degradation buffer (50 mM CHES at pH 10, 10 mM $MgCl_2$) in a final volume of 17.5 µL. After incubation at 37 °C for the indicated time points (0 min, 10 min, 20 min, 30 min, 45 min, 60 min, 120 min), 2.5 µL of the mixture were quenched with 0.5 M Tris-HCl (0.5 M, pH 7, 2.5 µL) and EDTA-Na (0.5 M, 1.5 µL) and stored at -80 °C. RNA integrity and length were confirmed by loading 200 ng mRNA onto 1 % denaturing agarose gel. Electrophoresis was performed for 80 min at 90 V in 1 × 3-(*N*-morpholino)propanesulfonic acid buffer and imaged on Typhoon FLA 9500 scanner (GE Healthcare).

For stability tests in 1 × PBS, 1.4 µg of mRNA was incubated in a final volume of 70 µL at 37 °C for up to 72h. At the indicated time points (0 h, 2 h, 4 h, 8 h, 24 h, 48 h, 72 h), 10 µL were removed and stored at -80 °C until gel electrophoresis was performed as described above.

PCR Amplification of Targeted Dual Fluorescence Reporter Plasmid

After flow cytometry, HEK293T cells were heated for 10 min at 95 °C. Then 5 µL of the supernatant was used for PCR amplification and send for sequencing.

Supplementary Information

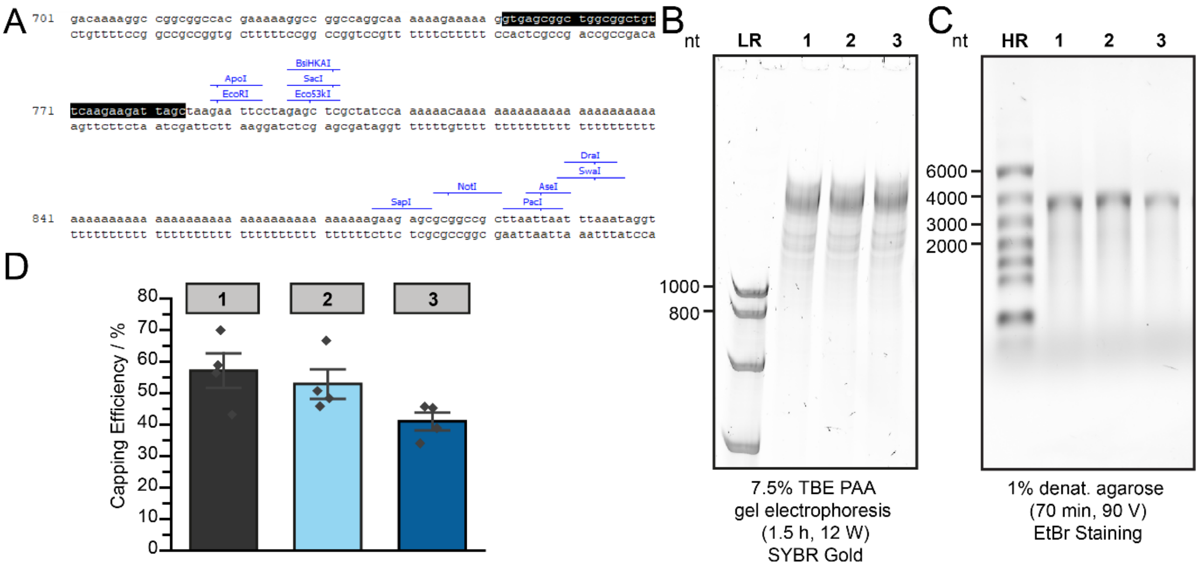

Supplementary Figure 1: A: Sequencing results for successful cloning of the C-terminal HiBiT-Tag (marked in black). B–C: Denaturing polyacrylamide gel electrophorese (PAGE) in 1 × Tris/Borate/EDTA buffer (left; 100 ng mRNA, 7.5% polyacrylamide gel, 1.5 h, 12 W) and denaturing agarose gel electrophorese in 1 × 3-(*N*-morpholino)propanesulfonic acid (MOPS) buffer (right; 500 ng mRNA, 70 min, 90 V). The Cas9-mRNA (with the HiBiT-tag) was analyzed after IVT. IVT was performed using the chemically synthesized NPM-FlashCap, cap0 or Ap$_3$G (Jena Bioscience). RiboRuler Low Range and RiboRuler High Range (Thermo Fisher Scientific) were used as a marker. D: Capping efficiency of HiBiT-tagged Cas9-mRNAs with respective cap analogs (**1**: m^{7}GpppG, **2**: ApppG, **3**: NPM-FlashCap). The capping efficiency is calculated by the quotient of the mRNA yield before digestion of uncapped mRNA and the mRNA yield after digestion.

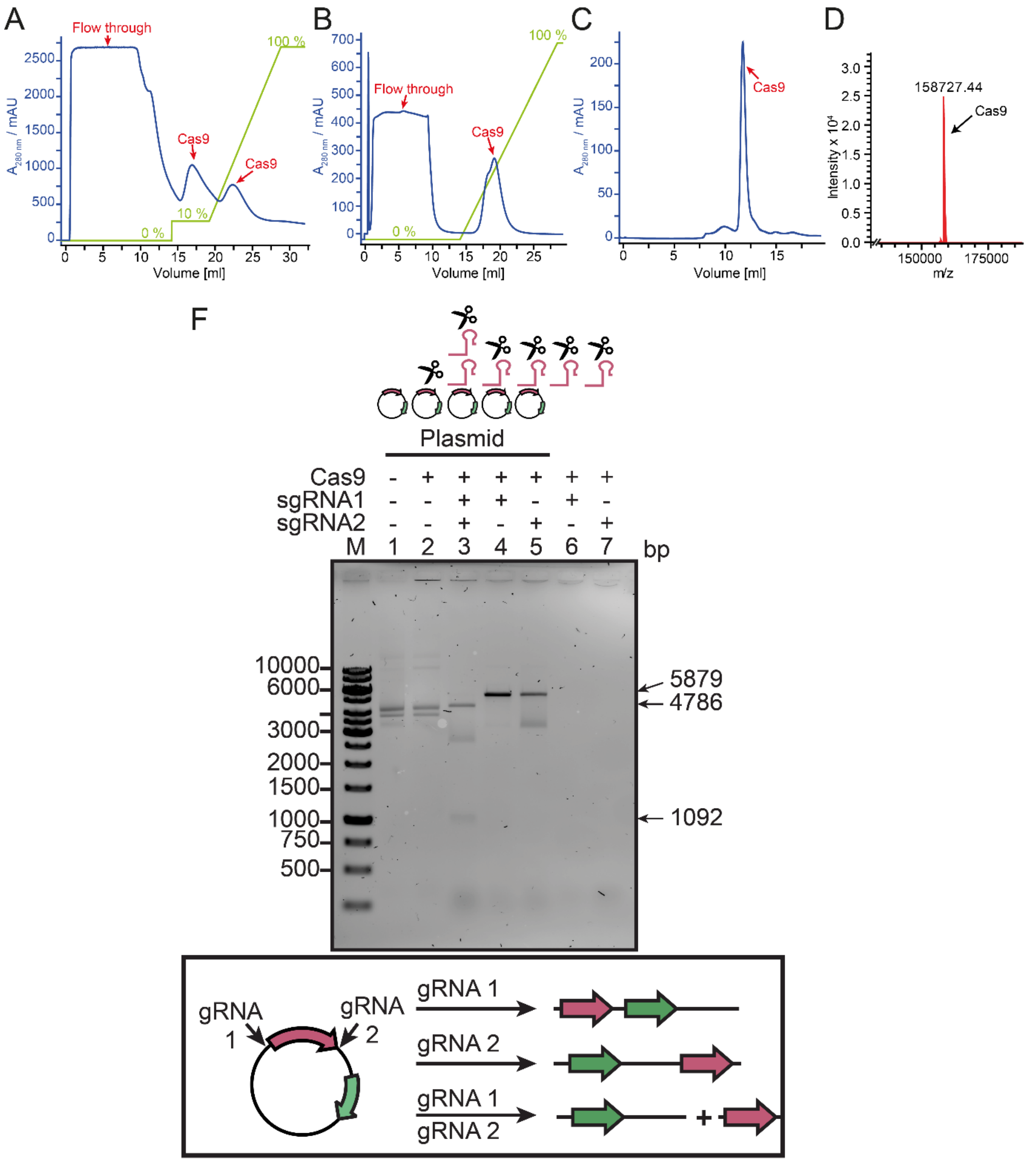

Supplementary Figure 2: A–E: AKTA purification chromatogram of Cas9 protein depicting the ratio of elution buffer [%] in green and the absorbance in mAU at 280 nm in blue after nickel affinity chromatography (A), ion exchange chromatography (B) and gel filtration (C). D: Deconvoluted protein mass with a peak at 158727.44 Da. F: *In vitro* cleavage assay with recombinantly produced Cas9 protein. Gene Ruler 1 kb DNA Ruler (Thermo Fisher Scientific) was used as a marker. Lane 1 (only plasmid), 2 (plasmid and Cas9), 6 (Cas9, sgRNA1) and 7 (Cas9, sgRNA2) served as controls. The dual fluorescence plasmid is cleaved twice due to the presence of both sgRNAs (lane 3) or once due to the presence of either sgRNA 1 (lane 4) or sgRNA 2 (lane 5).

Supplementary Table 4: Data for calculation of turn-on effect from main text figures. To calculate cap-dependent translation the value for ApppG-capped RNA was considered background and set to 0%, the value for m^7GpppG-capped RNA was set to 100%.

a) HiBit Cas9

	m7	m7 365 nm	Ap	Ap irr	NPM	NPM irr
	20812	17859	1018	541	2717	21439
	100%	86%	5%	3%	13%	103%
	19794	16841	0	-477	1699	20421
	100%	85%	0%	-2%	9%	103%
	10323	10032	477	187	2261	9008
	100%	97%	5%	2%	22%	87%
	9846	9555	0	-290	1784	8531
	100%	97%	0%	-3%	18%	87%
	14403	4928	913	489	2234	15405
	100%	34%	6%	3%	16%	107%
	13490	4015	0	-424	1321	14492
	100%	30%	0%	-3%	10%	107%
Mean value	100%	72%	5%	3%	17%	99%
Mean value	100%	71%	0%	-3%	12%	99%
Turn-On						8,1

b) HiBit RpS25-Cas9

	m7	m7 365 nm	Ap	Ap irr	NPM	NPM irr
	19156	13899	745	428	2245	12917
	100%	73%	4%	2%	12%	67%
ApppG substr	18411	13471	0	0	1500	12489
	100%	73%	0%	0%	8%	68%
	11784	9990	806	529	1099	4952
	100%	85%	7%	4%	9%	42%
ApppG substr	10978	9461	0	0	293	4423
	100%	86%	0%	0%	3%	40%
	36845	13266	918	515	2596	24408
	100%	36%	2%	1%	7%	66%
ApppG substr	35927	12751	0	0	1678	23893
	100%	35%	0%	0%	5%	67%
Mean value	100%	64%	4%	3%	9%	59%
Mean value	100%	65%	0%	0%	5%	58%
Turn-On						11,3

c) Flow cytometry Cas9

	300 ng						150 ng					
	m7	m7 365 nm	Ap	Ap irr	NPM	NPM irr	m7	m7 365 nm	Ap	Ap irr	NPM	NPM irr
eGFP	8797	10446	847	517	3049	6791	5938	7269	377	457	2250	6486
mScarlet	4993	7178	9523	8308	9021	7862	7178	9390	6662	8573	6791	8203
eGFP/mScarl	1,7618666	1,45528	0,0889426	0,0622292	0,3379891	0,8637751	0,8272499	0,7741214	0,0565896	0,0533069	0,3313209	0,7906863
	100%	83%	5%	4%	19%	49%	100%	94%	7%	6%	40%	96%
ApppG subst	1,6729241	1,3663375	0	-0,0267134	0,2490466	0,7748326	0,7706603	0,7175318	0	-0,0032827	0,2747313	0,7340967
	100%	82%	0%	-2%	15%	46%	100%	93%	0%	0%	36%	95%
	m7	m7 365 nm	Ap	Ap irr	NPM	NPM irr	m7	m7 365 nm	Ap	Ap irr	NPM	NPM irr
eGFP	11917	9895	2677	2820	7002	10546	24987	18507	1551	1255	11627	17975
mScarlet	1930	1938	5911	10952	5324	2405	9227	7672	13474	10584	14151	7202
eGFP/mScarl	6,1746114	5,1057792	0,4528845	0,2574872	1,3151766	4,3850312	2,7080308	2,4122784	0,1151106	0,1185752	0,821638	2,4958345
	100%	83%	7%	4%	21%	71%	100%	89%	4%	4%	30%	92%
ApppG subst	5,7217269	4,6528947	0	-0,1953972	0,8622921	3,9321467	2,5929202	2,2971678	0	0,0034646	0,7065275	2,3807239
	100%	81%	0%	-3%	15%	69%	100%	89%	0%	0%	27%	92%
	m7	m7 365 nm	Ap	Ap irr	NPM	NPM irr	m7	m7 365 nm	Ap	Ap irr	NPM	NPM irr
eGFP	53880	35307	4504	2601	16998	34098	38317	23987	2717	1401	12514	19619
mScarlet	15631	13702	22646	19454	21206	16062	17633	14435	25711	19988	21952	13459
eGFP/mScarl	3,4469964	2,5767771	0,1988872	0,1337	0,8015656	2,1228988	2,1730278	1,661725	0,1056746	0,0700921	0,570062	1,4576863
	100%	75%	6%	4%	23%	62%	100%	76%	5%	3%	26%	67%
ApppG subst	3,2481091	2,3778899	0	-0,0651872	0,6026784	1,9240115	2,0673532	1,5560504	0	-0,0355826	0,4643873	1,3520117
	100%	73%	0%	-2%	19%	59%	100%	75%	0%	-2%	22%	65%
Mean value	100%	80%	6%	4%	21%	61%	100%	86%	5%	5%	32%	85%
Mean value	100%	79%	0%	-2%	16%	58%	100%	86%	0%	-1%	28%	84%
Turn-On						3,5923786						2,957708

d) Flow cytometry RpS25-Cas9

	300 ng					
	m7	m7 365 nm	Ap	Ap irr	NPM	NPM irr
eGFP	1544	1093	24	16	207	568
mScarlet	1426	1145	1731	1206	1857	1162
eGFP/mScarl	1,08274895	0,95458515	0,01386482	0,013267	0,11147011	0,48881239
	100%	88%	1%	1%	10%	45%
ApppG subst	1,06888413	0,94072033	0	-0,0005978	0,0976053	0,47494757
	100%	88%	0%	0%	9%	44%
eGFP	5992	3846	138	79	870	3203
mScarlet	4271	4382	7446	7293	6202	5254
eGFP/mScarl	1,40295013	0,87768142	0,01853344	0,0108323	0,14027733	0,60963076
	100%	63%	1%	1%	10%	43%
ApppG subst	1,38441669	0,85914798	0	-0,0077011	0,12174389	0,59109732
	100%	62%	0%	-1%	9%	43%
eGFP	9430	4953	76	56	1082	3319
mScarlet	5015	4223	8306	5710	8663	4656
eGFP/mScarl	1,88035892	1,17286289	0,00915001	0,00980736	0,124899	0,71284364
	100%	62%	0%	1%	7%	38%
ApppG subst	1,87120891	1,16371288	0	0,00065734	0,11574898	0,70369363
	100%	62%	0%	0%	6%	38%
Mean value	100%	71%	1%	1%	9%	42%
Mean value	100%	71%	0%	0%	8%	42%
Turn-on						5,2

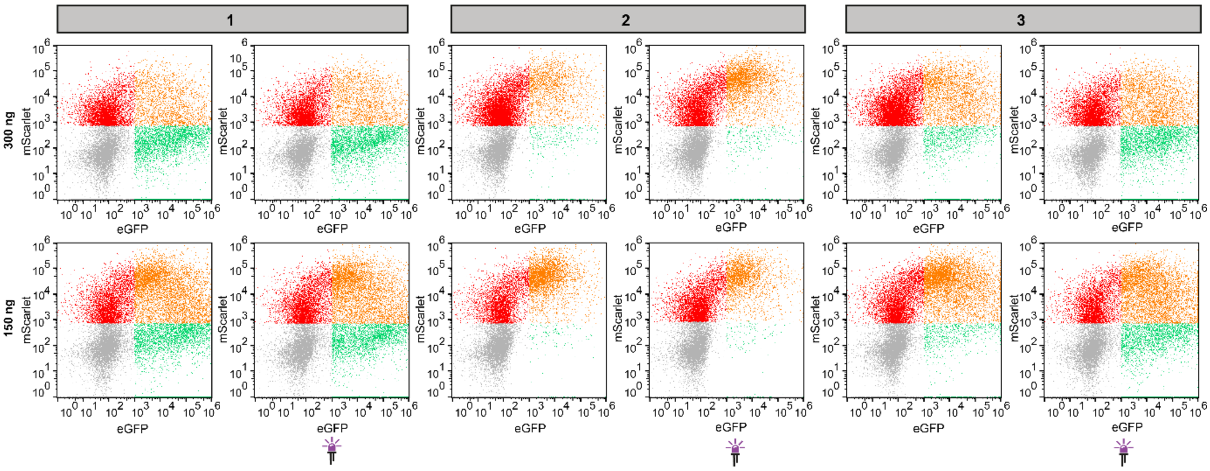

Supplementary Figure 3: Representative flow cytometry analysis showing dot plots of eGFP fluorescence and mScarlet fluorescence of HEK293T cells. HEK293T cells were first transfected with indicated amounts (300 ng, 150 ng) of differently capped Cas9 mRNAs (**1**: cap0, **2**: Ap$_3$G, **3**: NPM-FlashCap) and the two sgRNAs. Cells were left in the dark or irradiated after 4 h (violet LED: 365 nm, 10 s). After additional 4 h, cells were transfected with the dual-fluorescence reporter system.

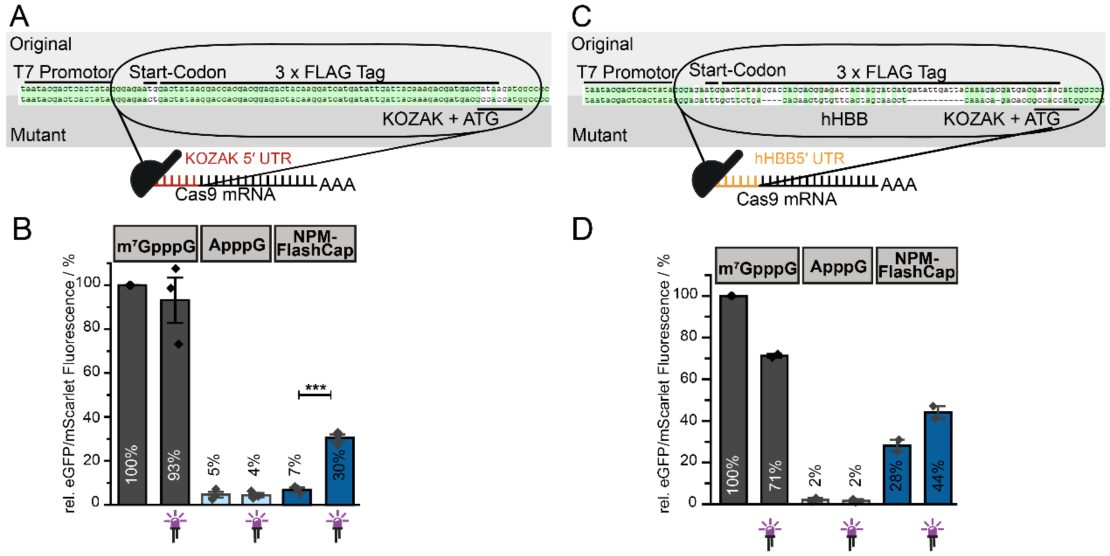

Supplementary Figure 4: A: The modified 5′ UTR (called Kozak 5′ UTR) and the comparison to the original construct. B: Relative eGFP/mScarlet fluorescence signal measured by flow cytometry. HEK293T cells were first transfected with different amounts (500 ng) of differently capped Cas9-mRNAs (construct described in A; m^{7}GpppG, ApppG, NPM-FlashCap) and the two sgRNAs. Cells were left in the dark or irradiated after 4 h (LED: 365 nm, 10 s). After additional 4 h, cells were transfected with the dual-fluorescence reporter system. Bars and error bars show mean value ±SEM of n=3 independent experiments. Statistical significance was determined by two-tailed Student's *t*-test. Significance levels were defined as *P < 0.05, **P < 0.01, ***P < 0.001. C: The modified construct with the hHBB 5′ UTR and the comparison to the original construct. D: Relative eGFP/mScarlet fluorescence signal measured by flow cytometry. HEK293T cells were first transfected with different amounts (500 ng) of differently capped Cas9-mRNAs (construct described in C; m^{7}GpppG, ApppG, NPM-FlashCap) and the two sgRNAs. Cells were left in the dark or irradiated after 4 h (LED: 365 nm, 10 s). After additional 4 h, cells were transfected with the dual-fluorescence reporter system. Bars and error bars show mean value ±SEM of n=3 independent experiments.

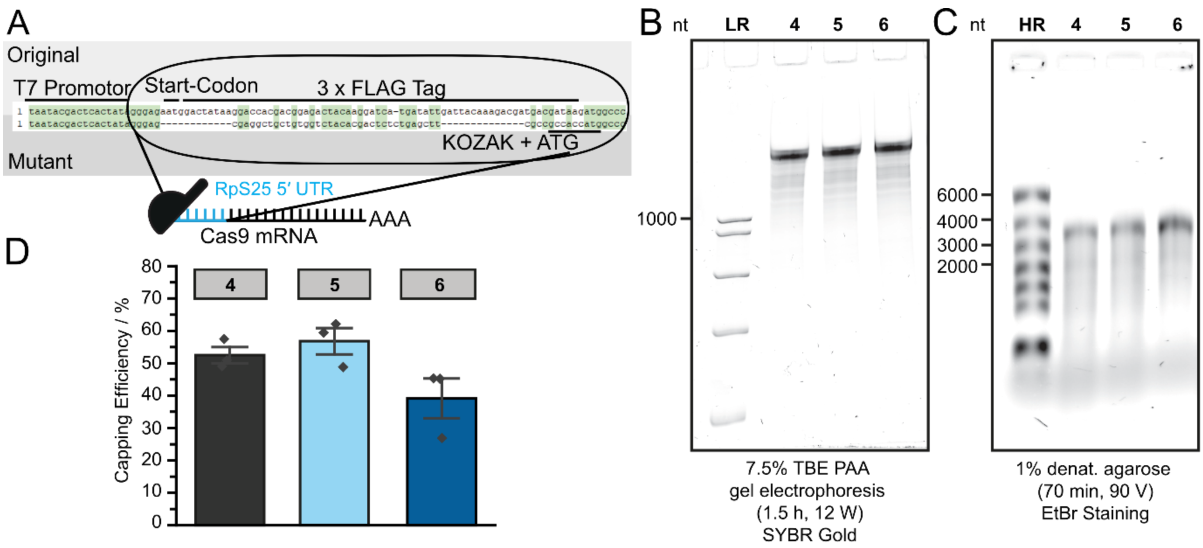

Supplementary Figure 5: A: Sequence of the original and new RpS25 5′ UTR construct (overlapping sequence is shown in green). B-C: Denaturing polyacrylamide gel electrophorese (PAGE) in 1 × Tris/Borate/EDTA buffer (left; 100 ng mRNA, 7.5% polyacrylamide gel, 1.5 h, 12 W) and denaturing agarose gel electrophorese in 1 × 3-(*N*-morpholino)propanesulfonic acid (MOPS) buffer (right; 500 ng mRNA, 70 min, 90 V). The Cas9-mRNA with the exchanged 5′ UTR was analyzed after IVT. IVT was performed using the chemically synthesized NPM-FlashCap (**6**), cap0 (**4**) or Ap$_3$G (**5**) (Jena Bioscience).RiboRuler Low Range (LR) and RiboRuler High Range (HR) (Thermo Fisher Scientific) were used as a marker. D: Capping efficiency of HiBiT-tagged Cas9-mRNAs with the exchanged 5′ UTR and respective cap analogs (**4**: m^{7}GpppG, **5**: ApppG, **6**: NPM-FlashCap). The capping efficiency is calculated by the quotient of the mRNA yield before digestion of uncapped mRNA and the mRNA yield after digestion.

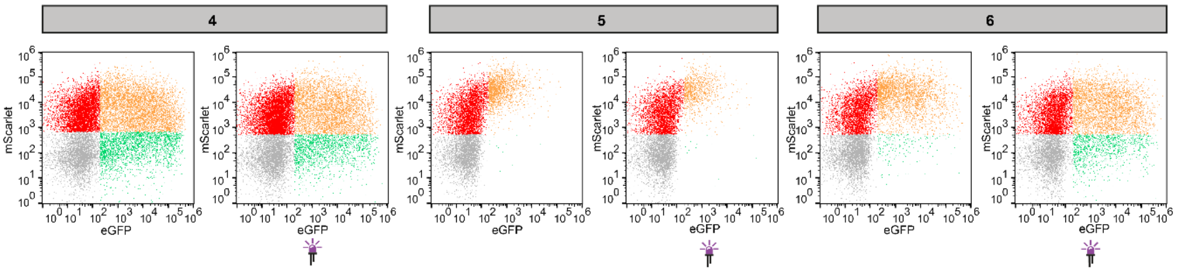

Supplementary Figure 6: Representative flow cytometry analysis showing dot plots of eGFP fluorescence and mScarlet fluorescence of HEK293T cells. HEK293T cells were first transfected with differently capped Cas9 mRNAs with the exchanged 5′ UTR (**4**: cap0, **5**: Ap$_3$G, **6**: NPM-FlashCap) and the two sgRNAs. Cells were left in the dark or irradiated after 4 h (violet LED: 365 nm, 10 s). After additional 4 h, cells were transfected with the dual-fluorescence reporter system.

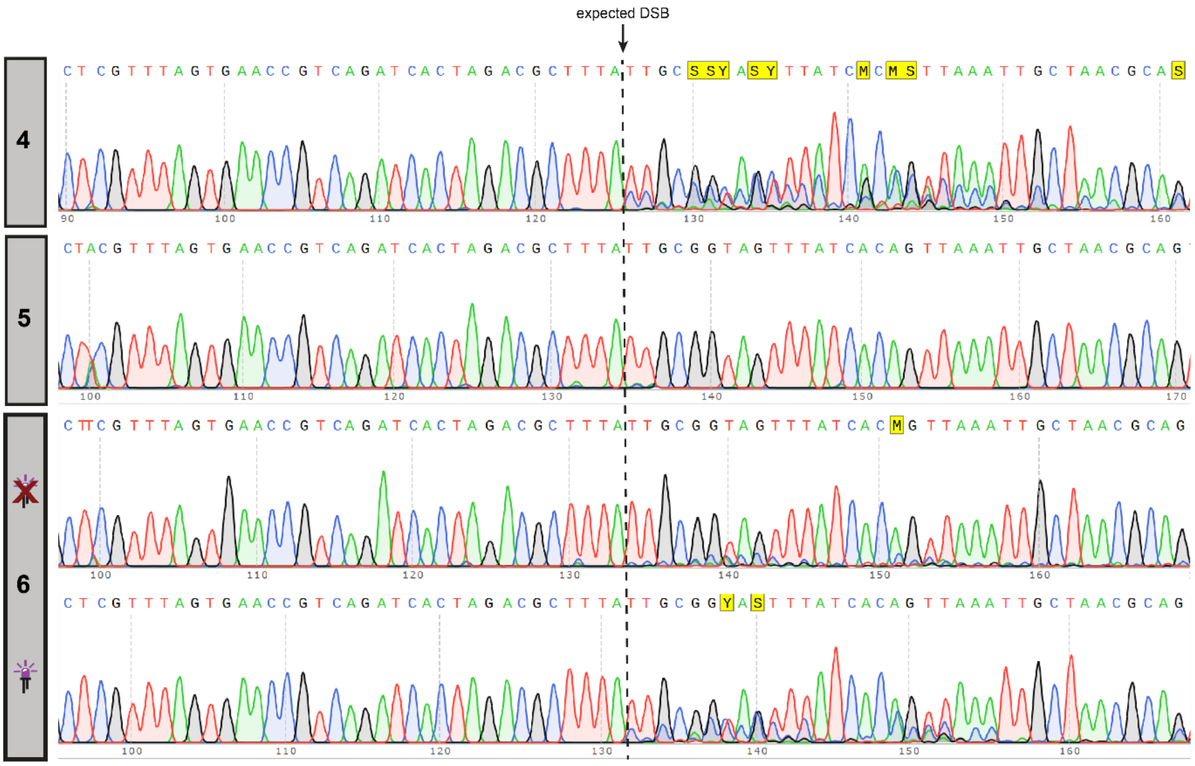

Supplementary Figure 7: Sanger Sequencing results of the PCR-amplified DNA templates from HEK293T cells transfected with differently capped Cas9 mRNAs with the exchanged 5′ UTR (**4**: cap0, **5**: Ap$_3$G, **6**: NPM-FlashCap with and without irradiation) and the two sgRNAs and the dual fluorescence reporter system.

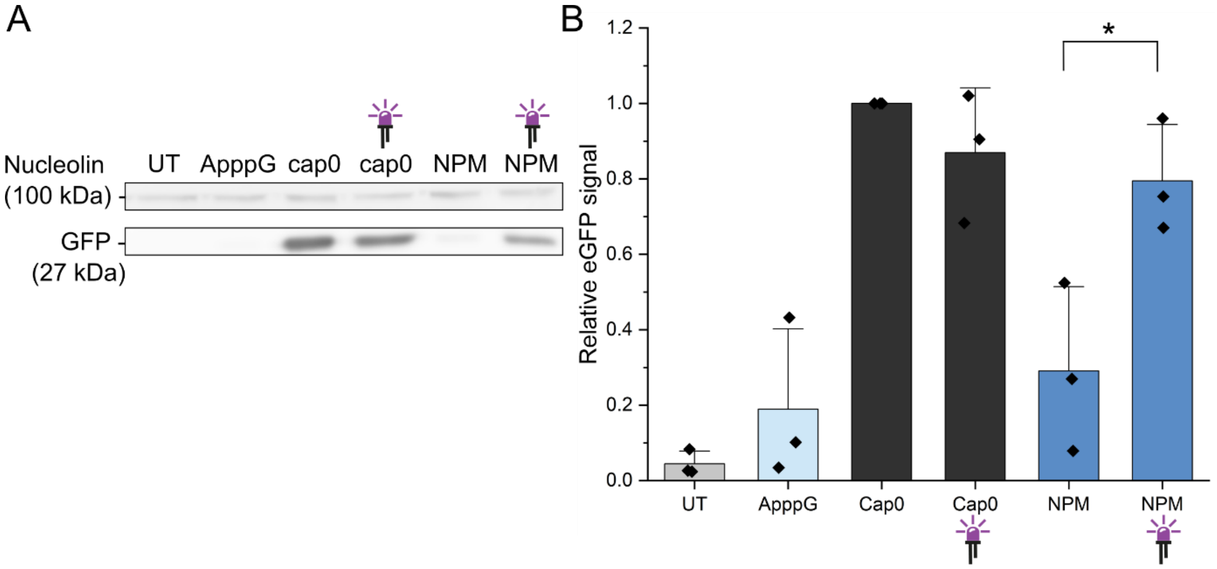

Supplementary Figure 8: The eGFP and Nucleolin protein levels analyzed via Western Blot. HEK293T cells were first transfected with differently capped RpS25-Cas9 mRNAs and the two sgRNAs. Cells were left in the dark or irradiated after 4 h (violet LED: 365 nm, 30 s). After additional 4 h, cells were transfected with the dual-fluorescence reporter system. Statistical significance was determined by two-tailed Student's t-test. Significance levels were defined as *P < 0.05, **P < 0.01, ***P < 0.001.

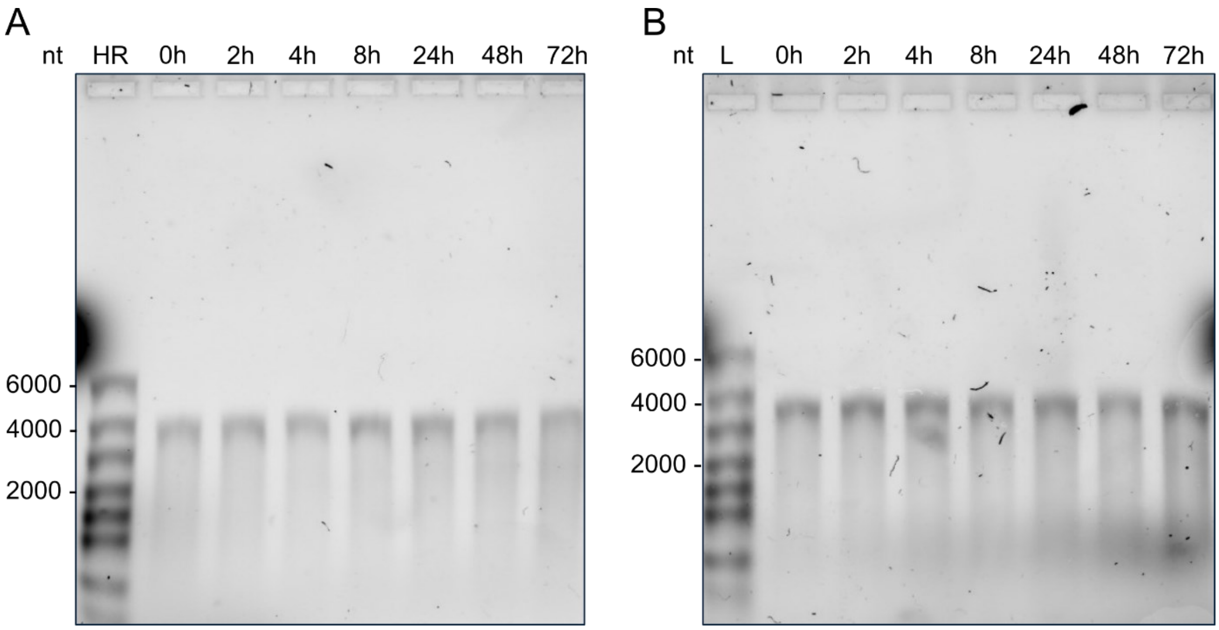

Supplementary Figure 9: Stability tests of differently capped mRNAs in 1 × PBS for up to 72 h. Denaturing agarose gel electrophorese in 1 × 3-(N-morpholino)propanesulfonic acid (MOPS) buffer (200 ng mRNA, 80 min, 90 V). A: Cap0-RpS25-Cas9 mRNA (**4**). B: NPM-RpS25-Cas9 mRNA (**6**). RiboRuler High Range (HR) (Thermo Fisher Scientific) was used as a marker.

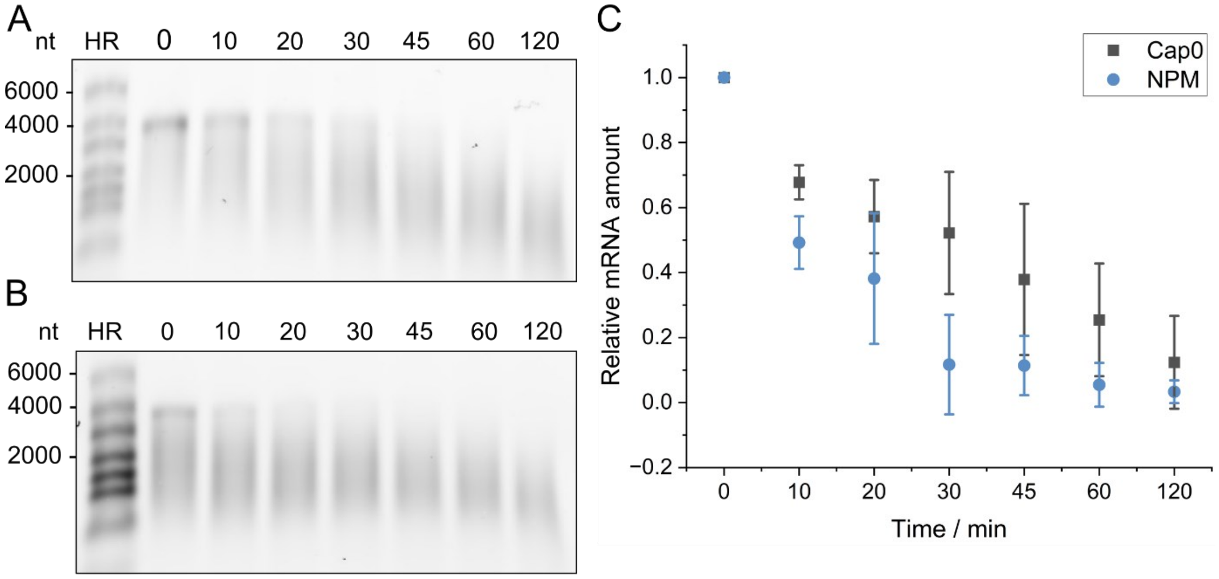

Supplementary Figure 10: Stability tests of differently capped mRNAs in degradation buffer (50 mM CHES at pH 10, 10 mM $MgCl_2$). A-B: Denaturing agarose gel electrophorese in 1 × 3-(N-morpholino)propanesulfonic acid (MOPS) buffer (200 ng mRNA, 80 min, 90 V). A: Cap0-RpS25-Cas9 mRNA. B: NPM- RpS25-Cas9 mRNA. RiboRuler High Range (HR) (Thermo Fisher Scientific) in degradation buffer was used as a marker. C: Remaining full length mRNA after the indicated time (n=3).

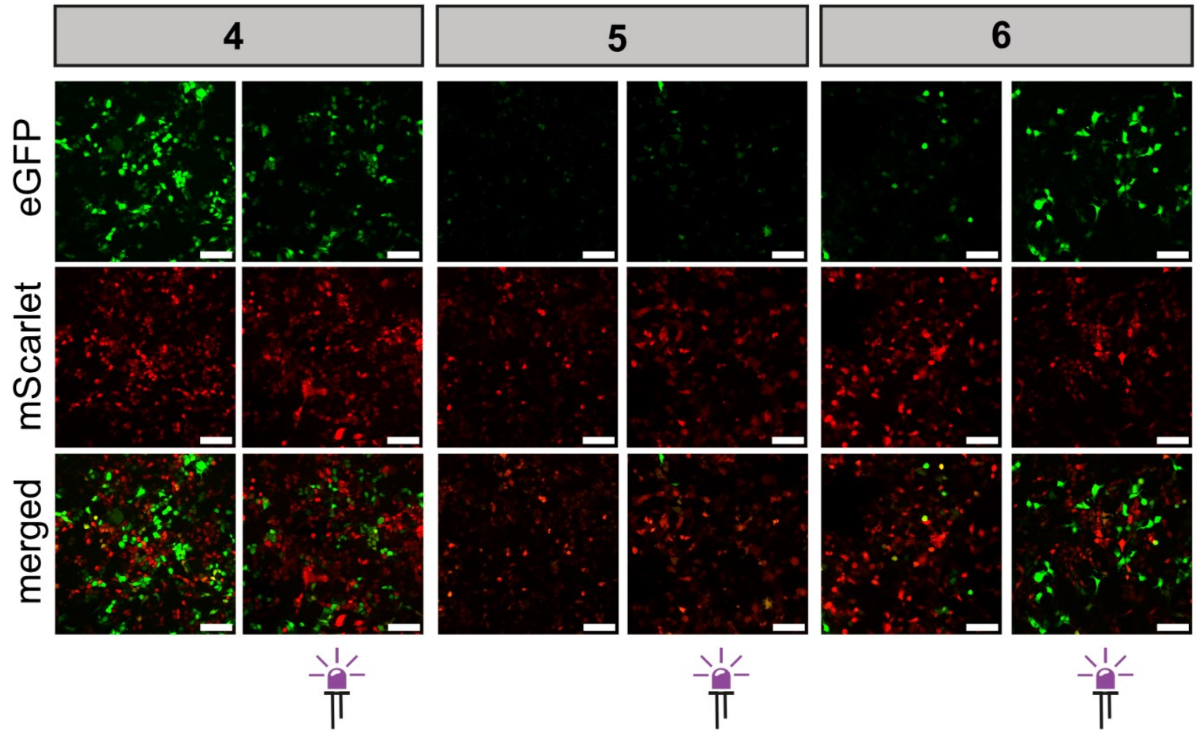

Supplementary Figure 11: Light-activated gene editing via photocaged RpS25-Cas9-mRNA in mammalian cells at higher resolution (complementing main text Figure 4). Microscopy images of HEK293T cells, transfected with differently capped Cas9-mRNAs (**1**: cap0, **2**: ApppG, **3**: NPM-FlashCap) and the two sgRNAs. Cells were left in the dark or irradiated after 4 h (LED: 365 nm, 10 s). After additional 4 h, cells were transfected with the dual-fluorescence reporter system. (scale bar = 100µm)

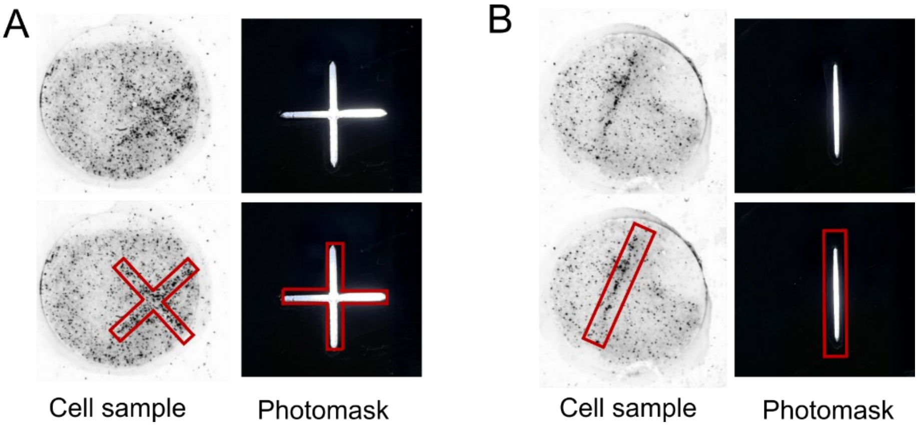

Supplementary Figure 12: Local activation of gene editing by photoactivation of RpS25-Cas9-mRNA. Side-to-side comparison of the irradiated cell sample and the photomask imaged on Typhoon FLA 9500 scanner (GE Healthcare) in the Cy2 (for eGFP) channel (complementing main text Figure 5).

Plasmid sequences

The 3xFLAG-Tag is shown in yellow, the NLS sequence is shown in green, the Cas9 sequence is shown in gray and the HiBiT-Tag is shown in turquoise: GTGAGCGGCTGGCGGCTGTTCAAGAAGATTAGC. The stop-codon is highlighted in red.

bbCas9pluspAAA+ HiBiT-Tag
TAATACGACTCACTATAGGGAGAATGGACTATAAGGACCACGACGGAGACTACAAGGAT<br>CATGATATTGATTACAAAGACGATGACGATAAGATGGCCCCAAAGAAGAAGCGGAAGG<br>TCGGTATCCACGGAGTCCCAGCAGCCGACAAGAAGTACAGCATCGGCCTGGACATCG<br>GCACCAACTCTGTGGGCTGGGCCGTGATCACCGACGAGTACAAGGTGCCCAGCAAG<br>AAATTCAAGGTGCTGGGCAACACCGACCGGCACAGCATCAAGAAGAACCTGATCGGA<br>GCCCTGCTGTTCGACAGCGGCGAAACAGCCGAGGCCACCCGGCTGAAGAGAACCGC<br>CAGAAGAAGATACACCAGACGGAAGAACCGGATCTGCTATCTGCAAGAGATCTTCAGC<br>AACGAGATGGCCAAGGTGGACGACAGCTTCTTCCACAGACTGGAAGAGTCCTTCCTG<br>GTGGAAGAGGATAAGAAGCACGAGCGGCACCCCATCTTCGGCAACATCGTGGACGAG<br>GTGGCCTACCACGAGAAGTACCCCACCATCTACCACCTGAGAAAGAAACTGGTGGAC<br>AGCACCGACAAGGCCGACCTGCGGCTGATCTATCTGGCCCTGGCCCACATGATCAAG<br>TTCCGGGGCCACTTCCTGATCGAGGGCGACCTGAACCCCGACAACAGCGACGTGGA<br>CAAGCTGTTCATCCAGCTGGTGCAGACCTACAACCAGCTGTTCGAGGAAAACCCCAT<br>CAACGCCAGCGGCGTGGACGCCAAGGCCATCCTGTCTGCCAGACTGAGCAAGAGCA<br>GACGGCTGGAAAATCTGATCGCCCAGCTGCCCGGCGAGAAGAAGAATGGCCTGTTC<br>GGAAACCTGATTGCCCTGAGCCTGGGCCTGACCCCCAACTTCAAGAGCAACTTCGAC<br>CTGGCCGAGGATGCCAAACTGCAGCTGAGCAAGGACACCTACGACGACGACCTGGA<br>CAACCTGCTGGCCCAGATCGGCGACCAGTACGCCGACCTGTTTCTGGCCGCCAAGAA<br>CCTGTCCGACGCCATCCTGCTGAGCGACATCCTGAGAGTGAACACCGAGATCACCAA<br>GGCCCCCCTGAGCGCCTCTATGATCAAGAGATACGACGAGCACCACCAGGACCTGAC<br>CCTGCTGAAAGCTCTCGTGCGGCAGCAGCTGCCTGAGAAGTACAAAGAGATTTTCTT<br>CGACCAGAGCAAGAACGGCTACGCCGGCTACATTGACGGCGGAGCCAGCCAGGAAG<br>AGTTCTACAAGTTCATCAAGCCCATCCTGGAAAAGATGGACGGCACCGAGGAACTGCT<br>CGTGAAGCTGAACAGAGAGGACCTGCTGCGGAAGCAGCGGACCTTCGACAACGGCA<br>GCATCCCCCACCAGATCCACCTGGGAGAGCTGCACGCCATTCTGCGGCGGCAGGAA<br>GATTTTTACCCATTCCTGAAGGACAACCGGGAAAAGATCGAGAAGATCCTGACCTTCC<br>GCATCCCCTACTACGTGGGCCCTCTGGCCAGGGGAAACAGCAGATTCGCCTGGATGA<br>CCAGAAAGAGCGAGGAAACCATCACCCCCTGGAACTTCGAGGAAGTGGTGGACAAG<br>GGCGCTTCCGCCCAGAGCTTCATCGAGCGGATGACCAACTTCGATAAGAACCTGCCC<br>AACGAGAAGGTGCTGCCCAAGCACAGCCTGCTGTACGAGTACTTCACCGTGTATAAC<br>GAGCTGACCAAAGTGAAATACGTGACCGAGGGAATGAGAAAGCCCGCCTTCCTGAGC

GGCGAGCAGAAAAAGGCCATCGTGGACCTGCTGTTCAAGACCAACCGGAAAGTGAC
CGTGAAGCAGCTGAAAGAGGACTACTTCAAGAAAATCGAGTGCTTCGACTCCGTGGA
AATCTCCGGCGTGGAAGATCGGTTCAACGCCTCCCTGGGCACATACCACGATCTGCT
GAAAATTATCAAGGACAAGGACTTCCTGGACAATGAGGAAAACGAGGACATTCTGGAA
GATATCGTGCTGACCCTGACACTGTTTGAGGACAGAGAGATGATCGAGGAACGGCTG
AAAACCTATGCCCACCTGTTCGACGACAAAGTGATGAAGCAGCTGAAGCGGCGGAGA
TACACCGGCTGGGGCAGGCTGAGCCGGAAGCTGATCAACGGCATCCGGGACAAGCA
GTCCGGCAAGACAATCCTGGATTTCCTGAAGTCCGACGGCTTCGCCAACAGAAACTT
CATGCAGCTGATCCACGACGACAGCCTGACCTTTAAAGAGGACATCCAGAAAGCCCA
GGTGTCCGGCCAGGGCGATAGCCTGCACGAGCACATTGCCAATCTGGCCGGCAGCC
CCGCCATTAAGAAGGGCATCCTGCAGACAGTGAAGGTGGTGGACGAGCTCGTGAAAG
TGATGGGCCGGCACAAGCCCGAGAACATCGTGATCGAAATGGCCAGAGAGAACCAGA
CCACCCAGAAGGGACAGAAGAACAGCCGCGAGAGAATGAAGCGGATCGAAGAGGGC
ATCAAAGAGCTGGGCAGCCAGATCCTGAAAGAACACCCCGTGGAAAACACCCAGCTG
CAGAACGAGAAGCTGTACCTGTACTACCTGCAGAATGGGCGGGATATGTACGTGGACC
AGGAACTGGACATCAACCGGCTGTCCGACTACGATGTGGACCATATCGTGCCTCAGA
GCTTTCTGAAGGACGACTCCATCGACAACAAGGTGCTGACCAGAAGCGACAAGAACC
GGGGCAAGAGCGACAACGTGCCCTCCGAAGAGGTCGTGAAGAAGATGAAGAACTAC
TGGCGGCAGCTGCTGAACGCCAAGCTGATTACCCAGAGAAAGTTCGACAATCTGACC
AAGGCCGAGAGAGGCGGCCTGAGCGAACTGGATAAGGCCGGCTTCATCAAGAGACA
GCTGGTGGAAACCCGGCAGATCACAAAGCACGTGGCACAGATCCTGGACTCCCGGAT
GAACACTAAGTACGACGAGAATGACAAGCTGATCCGGGAAGTGAAAGTGATCACCCT
GAAGTCCAAGCTGGTGTCCGATTTCCGGAAGGATTTCCAGTTTTACAAAGTGCGCGAG
ATCAACAACTACCACCACGCCCACGACGCCTACCTGAACGCCGTCGTGGGAACCGCC
CTGATCAAAAAGTACCCTAAGCTGGAAAGCGAGTTCGTGTACGGCGACTACAAGGTGT
ACGACGTGCGGAAGATGATCGCCAAGAGCGAGCAGGAAATCGGCAAGGCTACCGCC
AAGTACTTCTTCTACAGCAACATCATGAACTTTTTCAAGACCGAGATTACCCTGGCCAA
CGGCGAGATCCGGAAGCGGCCTCTGATCGAGACAAACGGCGAAACCGGGGAGATCG
TGTGGGATAAGGGCCGGGATTTTGCCACCGTGCGGAAAGTGCTGAGCATGCCCCAAG
TGAATATCGTGAAAAAGACCGAGGTGCAGACAGGCGGCTTCAGCAAAGAGTCTATCCT
GCCCAAGAGGAACAGCGATAAGCTGATCGCCAGAAAGAAGGACTGGGACCCTAAGAA
GTACGGCGGCTTCGACAGCCCCACCGTGGCCTATTCTGTGCTGGTGGTGGCCAAAGT
GGAAAAGGGCAAGTCCAAGAAACTGAAGAGTGTGAAAGAGCTGCTGGGGATCACCAT
CATGGAAAGAAGCAGCTTCGAGAAGAATCCCATCGACTTTCTGGAAGCCAAGGGCTA
CAAAGAAGTGAAAAAGGACCTGATCATCAAGCTGCCTAAGTACTCCCTGTTCGAGCTG
GAAAACGGCCGGAAGAGAATGCTGGCCTCTGCCGGCGAACTGCAGAAGGGAAACGA
ACTGGCCCTGCCCTCCAAATATGTGAACTTCCTGTACCTGGCCAGCCACTATGAGAAG

CTGAAGGGCTCCCCCGAGGATAATGAGCAGAAACAGCTGTTTGTGGAACAGCACAAG
CACTACCTGGACGAGATCATCGAGCAGATCA
GCGAGTTCTCCAAGAGAGTGATCCTGGCCGACGCTAATCTGGACAAAGTGCTGTCCG
CCTACAACAAGCACCGGGATAAGCCCATCAGAGAGCAGGCCGAGAATATCATCCACCT
GTTTACCCTGACCAATCTGGGAGCCCCTGCCGCCTTCAAGTACTTTGACACCACCATC
GACCGGAAGAGGTACACCAGCACCAAAGAGGTGCTGGACGCCACCCTGATCCACCA
GAGCATCACCGGCCTGTACGAGACACGGATCGACCTGTCTCAGCTGGGAGGCGACA
AAAGGCCGGCGGCCACGAAAAAGGCCGGCCAGGCAAAAAAGAAAAAGGTGAGCGG
CTGGCGGCTGTTCAAGAAGATTAGCTAAGAATTCCTAGAGCTCGCTAAAAAAAAAAAAA
AAAAAAAAAAAAAAAAAAAAAAAAAAAAAAAAAAAAAAAAAAAAAAAAAAAAAAAAAAAA
AAAAAAAAAAAAAAAAAAAAAAAAAAAAAAGAAGAGCGCGGCCGCTTAATTAATTTAAAT
AGGTGGCACTTTTCGGGGAAATGTGCGCGGAACCCCTATTTGTTTATTTTTCTAAATAC
ATTCAAATATGTATCCGCTCATGAGACAATAACCCTGATAAATGCTTCAATAATATTGAAA
AAGGAAGAGTATGAGTATTCAACATTTCCGTGTCGCCCTTATTCCCTTTTTTGCGGCAT
TTTGCCTTCCTGTTTTTGCTCACCCAGAAACGCTGGTGAAAGTAAAAGATGCTGAAGA
TCAGTTGGGTGCACGAGTGGGTTACATCGAACTGGATCTCAACAGCGGTAAGATCCTT
GAGAGTTTTCGCCCCGAAGAACGTTTTCCAATGATGAGCACTTTTAAAGTTCTGCTATG
TGGCGCGGTATTATCCCGTATTGACGCCGGGCAAGAGCAACTCGGTCGCCGCATACA
CTATTCTCAGAATGACTTGGTTGAGTACTCACCAGTCACAGAAAAGCATCTTACGGATG
GCATGACAGTAAGAGAATTATGCAGTGCTGCCATAACCATGAGTGATAACACTGCGGC
CAACTTACTTCTGACAACGATCGGAGGACCGAAGGAGCTAACCGCTTTTTTGCACAAC
ATGGGGGATCATGTAACTCGCCTTGATCGTTGGGAACCGGAGCTGAATGAAGCCATAC
CAAACGACGAGCGTGACACCACGATGCCTGTAGCAATGGCAACAACGTTGCGCAAAC
TATTAACTGGCGAACTACTTACTCTAGCTTCCCGGCAACAATTAATAGACTGGATGGAG
GCGGATAAAGTTGCAGGACCACTTCTGCGCTCGGCCCTTCCGGCTGGCTGGTTTATT
GCTGATAAATCTGGAGCCGGTGAGCGTGGGTCTCGCGGTATCATTGCAGCACTGGGG
CCAGATGGTAAGCCCTCCCGTATCGTAGTTATCTACACGACGGGGAGTCAGGCAACTA
TGGATGAACGAAATAGACAGATCGCTGAGATAGGTGCCTCACTGATTAAGCATTGGTAA
CTGTCAGACCAAGTTTACTCATATATACTTTAGATTGATTTAAAACTTCATTTTTAATTTAA
AAGGATCTAGGTGAAGATCCTTTTTGATAATCTCATGACCAAAATCCCTTAACGTGAGTT
TTCGTTCCACTGAGCGTCAGACCCCGTAGAAAAGATCAAAGGATCTTCTTGAGATCCT
TTTTTTCTGCGCGTAATCTGCTGCTTGCAAACAAAAAAACCACCGCTACCAGCGGTGG
TTTGTTTGCCGGATCAAGAGCTACCAACTCTTTTTCCGAAGGTAACTGGCTTCAGCAG
AGCGCAGATACCAAATACTGTTCTTCTAGTGTAGCCGTAGTTAGGCCACCACTTCAAGA
ACTCTGTAGCACCGCCTACATACCTCGCTCTGCTAATCCTGTTACCAGTGGCTGCTGC
CAGTGGCGATAAGTCGTGTCTTACCGGGTTGGACTCAAGACGATAGTTACCGGATAAG
GCGCAGCGGTCGGGCTGAACGGGGGGTTCGTGCACACAGCCCAGCTTGGAGCGAA

CGACCTACACCGAACTGAGATACCTACAGCGTGAGCTATGAGAAAGCGCCACGCTTC<br>CCGAAGGGAGAAAGGCGGACAGGTATCCGGTAAGCGGCAGGGTCGGAACAGGAGA<br>GCGCACGAGGGAGCTTCCAGGGGGAAACGCCTGGTATCTTTATAGTCCTGTCGGGTT<br>TCGCCACCTCTGACTTGAGCGTCGATTTTTGTGATGCTCGTCAGGGGGGCGGAGCCT<br>ATGGAAAAACGCCAGCAACGCGGCCTTTTTACGGTTCCTGGCCTTTTGCTGGCCTTTT<br>GCTCACATGTTCTTTCCTGCGTTATCCCCTGATTCTGTGGATAACCGTATTACCGCCTTT<br>GAGTGAGCTGATACCGCTCGCCGCAGCCGAACGACCGAGCGCAGCGAGTCAGTGAG<br>CGAGGAAGCGGAAGACCTGCAGGACCCATCTAGA
bbCas9pluspAAA + 5′ UTR + HiBiT-Tag which is shown until the NLS sequence
TAATACGACTCACTATAGGGAGCGAGGCTGCTGTGGTCTACACGACTCTCTGAGCTTC<br>GCCGCCACCATGGCCCCAAAGAAGAAGCGGAAGGTC

References

1. Klöcker, N.; Weissenboeck, F. P.; van Dülmen, M.; Špaček, P.; Hüwel, S.; Rentmeister, A., Photocaged 5′ cap analogues for optical control of mRNA translation in cells. *Nat. Chem.* **2022,** *14* (8), 905–913.
2. Williams, D. J.; Puhl III, H. L.; Ikeda, S. R., A simple, highly efficient method for heterologous expression in mammalian primary neurons using cationic lipid-mediated mRNA transfection. *Frontiers in neuroscience* **2010,** *4*, 181.
3. Jinek, M.; Chylinski, K.; Fonfara, I.; Hauer, M.; Doudna, J. A.; Charpentier, E., A programmable dual-RNA–guided DNA endonuclease in adaptive bacterial immunity. *Science* **2012,** *337* (6096), 816–821.
4. Henriksson, J.; Chen, X.; Gomes, T.; Ullah, U.; Meyer, K. B.; Miragaia, R.; Duddy, G.; Pramanik, J.; Yusa, K.; Lahesmaa, R., Genome-wide CRISPR screens in T helper cells reveal pervasive crosstalk between activation and differentiation. *Cell* **2019,** *176* (4), 882–896. e18.
5. Pritchard, C. E.; Kroese, L. J.; Huijbers, I. J., Direct generation of conditional alleles using CRISPR/Cas9 in mouse zygotes. *Site-Specific Recombinases: Methods and Protocols* **2017,** *1642*, 21–35.
